# Sex-Stratified Characterization of Behavioral, Hippocampal, and Microglial Androgen Responses to Adolescent High-Fat Diet Consumption in Mice

**DOI:** 10.3390/nu18193229

**Published:** 2026-09-30

**Authors:** Conghui Wang, Hong Li, Zhehao Yu, Bingjie Xie, Xiuting Yao, Jingyi Zhao, Yuxi Chen, Hong Zhuang, Chongyang Yang, Lijie Liu

**Affiliations:** 1 Medical College, Southeast University, Nanjing 210009, China; jnconghui2009@126.com (C.W.); 17551120513@163.com (Z.Y.); 220234159@seu.edu.cn (B.X.); delia415171022@163.com (X.Y.); 230239144@seu.edu.cn (Y.C.); 101300426@seu.edu.cn (H.Z.); 220244350@seu.edu.cn (C.Y.); 2 School of Life Science and Technology, Southeast University, Nanjing 210009, China; 13203929028@163.com (H.L.); zjysk175@163.com (J.Z.); 3 Jiangsu Provincial Key Laboratory of Critical Care Medicine, Department of Physiology, School of Medicine, Southeast University, Nanjing 210009, China

**Keywords:** sex stratification, adolescence, depression, high-fat diet, microglia, androgen

## Abstract

**Background**: Adolescence is a vulnerable developmental period during which dietary exposure and psychosocial stressors may have lasting effects on higher-order brain functions. Depression often emerges during adolescence, at which time a female preponderance in prevalence becomes apparent. Consumption of a high-fat diet (HFD) is common during adolescence and has been associated with adverse emotional and neurobiological outcomes; however, its consequences have not been systematically characterized in both sexes. **Methods**: Male and female mice were fed a HFD from postnatal days 28 to 56 (spanning most of adolescence), with chronic unpredictable mild stress (CUMS) used as a challenge to assess stress responsiveness. **Results**: Sex-stratified analyses revealed that HFD consumption induced anhedonia-like behavior in both sexes, whereas additional behavioral impairments were detected in females. Additionally, HFD consumption amplified the behavioral response to CUMS in females. HFD feeding altered the ventral hippocampal neurostructure and microglial status in both sexes; however, dendritic and spine deficits and microglial activation-like changes were detected across more ventral hippocampal subregions in females, together with increased synaptic engulfment in the vDG. HFD consumption increased circulating leptin and estradiol levels in both sexes, and higher free fatty acid levels were detected in nonstressed HFD-fed mice of both sexes. In females, HFD consumption increased circulating testosterone levels, which was accompanied by increased androgen receptor (AR) nuclear localization and TNF-α expression in hippocampal microglia. Serum from HFD-fed females significantly increased AR nuclear localization (*p* = 0.0008) and phagocytic activity (*p* = 0.0038) in primary female microglia. Enzalutamide abolished the serum-induced increase in AR nuclear localization (*p* < 0.0001), reduced the corresponding increase in phagocytic activity (*p* = 0.0335), and attenuated the associated induction of TNF-α at both the protein (*p* = 0.0057) and mRNA (*p* = 0.0003) levels. **Conclusions**: These findings characterize sex-stratified behavioral and hippocampal neurobiological consequences of adolescent HFD consumption and suggest a previously underrecognized association between androgen-responsive microglial signaling and the broader hippocampal alterations observed in females. Although the causal contribution of this signaling pathway to the in vivo phenotypes remains to be established, this work provides a framework for further investigating sex-related differences in the neurobiological consequences of adolescent HFD consumption and the potential contribution of endocrine–microglial signaling.

## 1. Introduction

Depression is a multifactorial disorder shaped by complex interactions among environmental, metabolic, endocrine, and neuroimmune factors [1,2]. A striking feature of depression is its pronounced sex bias, with the incidence increasing disproportionately in females after adolescence [3]. Adolescence represents a sensitive developmental period characterized by extensive brain maturation, endocrine transition, and heightened responsiveness to environmental influences [1,4,5]. During this period, dietary habits also undergo substantial changes, including increased consumption of energy-dense, high-fat foods [6]. Emerging evidence indicates that excessive high-fat diet (HFD) consumption during adolescence can adversely affect emotional behavior and brain plasticity [7,8]; however, the extent to which these effects differ between males and females remains incompletely characterized.

Accumulating evidence indicates sex-dependent metabolic, endocrine, and neuroimmune responses to HFD exposure, with the direction and magnitude of these effects varying across experimental conditions [9,10,11,12]. Recent work further revealed that consumption of a HFD in adolescence alters microglial transcriptional responses to subsequent challenges in a sex-dependent manner, including the differential regulation of inflammatory genes in male and female mice [13]. Chronic stress is another major environmental factor related to depression [14], and emerging evidence suggests that metabolic disturbances may interact with stress exposure to shape behavioral and neuroimmune outcomes [15]. These observations raise the possibility that adolescent HFD consumption alters the magnitude or pattern of subsequent stress-related responses in a sex-dependent manner. Accordingly, rather than serving as a primary etiological model, chronic unpredictable mild stress (CUMS) [16] was used here as a functional challenge to determine whether HFD exposure affects stress-related behavioral disturbances.

The hippocampus is particularly vulnerable to metabolic and environmental perturbations during adolescence and plays essential roles in emotional regulation [5,17]. Its dorsal and ventral subdivisions are functionally differentiated, with the ventral hippocampus (vHP) being more strongly involved in affective processing [18]. Structural abnormalities within hippocampal subregions have been associated with depression-related phenotypes and stress responsiveness [19,20]. Previous studies have shown that a HFD can disrupt hippocampal plasticity in both sexes, although the affected cellular processes and hippocampal subregions may vary with sex, developmental stage, and dietary paradigm. For example, chronic HFD consumption has been reported to impair the dendritic morphology of hippocampal CA1 pyramidal neurons in males [21], whereas HFD-induced obesity reduces the number of immature neurons in the dentate gyrus (DG) of females [22]. Our previous work demonstrated that adolescent HFD consumption induces depression-like behaviors and hippocampal neurostructural alterations in male mice, effects that were attenuated by the microglial inhibitor minocycline [8,23], supporting a contributory role for aberrant microglial activation in HFD-associated hippocampal remodeling. As key regulators of synaptic remodeling, neuroimmune signaling, and stress adaptation, microglia are themselves shaped by sex, developmental stage, brain region, and physiological context [24,25,26]. Recent evidence further indicates that adolescent HFD consumption can elicit sex-dependent microglial transcriptional responses and differentially modify their responses to subsequent stressors [13]. Together, these findings position microglia as a potential cellular interface through which sex-dependent systemic responses to adolescent HFD consumption may influence hippocampal plasticity.

Microglia are highly responsive to changes in the circulating metabolic and endocrine milieu [27]. Free fatty acids (FFAs) [8,28,29], metabolic hormones such as leptin [30], and gonadal steroids [25,31] can modulate microglial inflammatory and phagocytic functions. This is particularly relevant during adolescence, when marked developmental changes in metabolic and gonadal hormone signaling coincide with the ongoing maturation of the neural and immune systems [1,32,33], and these circulating signals can themselves be altered by HFD exposure [34,35]. Sex steroid signaling may be especially pertinent during adolescence because gonadal hormone levels undergo substantial developmental changes, whereas obesogenic dietary exposure can further perturb the reproductive endocrine milieu in a sex-dependent manner [36,37]. Among these pathways, androgen signaling has been shown to modulate microglial phenotype and immune function, although the direction and magnitude of these effects vary with sex and experimental context [31,38]. Thus, the specific circulating signals and downstream pathways that contribute to sex-dependent microglial responses to adolescent HFD consumption remain to be defined.

In this study, we performed a sex-stratified analysis of behavioral outcomes, circulating levels of metabolic and endocrine factors, hippocampal neurostructure, and microglial status in male and female mice exposed to a HFD during adolescence, with CUMS employed as a secondary challenge to probe stress responsiveness. We then used the sex-specific circulating and microglial alterations identified in vivo to guide mechanistic follow-up in primary hippocampal microglia, including pharmacological interrogation of signaling pathways emerging from these analyses. This approach enabled us to characterize sex-dependent neurobehavioral and hippocampal consequences of adolescent HFD consumption and to identify candidate circulating-to-microglial mechanisms that may contribute to the broader alterations observed in females.

## 2. Materials and Methods

### 2.1. Animals

Male and female C57BL/6 mice (*n* = 72 total, 36 males and 36 females), aged 21 days (P21), were purchased from Changzhou Cavens Experimental Animal Co., Ltd. (Changzhou, China, SCXK (SU) 2021-0013) and were group-housed (three mice per cage) in same-sex cages under standard conditions (lights on from 7 a.m. to 7 p.m., 22 °C, and 55% humidity) with food and water available ad libitum. After one week of acclimatization, the mice of each sex were randomly assigned to either the standard diet (SD) group or the HFD group (*n* = 18) and were fed their respective diets for four weeks (P28–P56, spanning most of the adolescence period [39]). Body weight was recorded weekly. Cage-level food consumption, expressed as the amount of food consumed by weight, was measured every four days. The average daily caloric intake per animal was calculated from the amount of food consumed, the energy density of the respective diet, the number of mice per cage, and the measurement interval. Body weight gain was determined as the difference between body weight at P56 and P28. After two weeks of dietary treatment (on P42), each diet group within each sex was further randomly divided into control (Ctrl) and CUMS subgroups (*n* = 9 per subgroup), and the assigned diets were continued. The mice in the CUMS subgroups were then subjected to the CUMS protocol, whereas the Ctrl mice remained group-housed under standard conditions without exposure to CUMS, which is consistent with the control conditions commonly used in CUMS studies [40]. The mice remained on their assigned diets continuously throughout the entire experimental period until they were euthanized. At the end of the experiment, brain tissue was collected for morphological analyses, and serum samples were collected for biochemical measurements or treatment of primary microglia in vitro. All the animal experiments were conducted according to protocols approved by the University Committee for Laboratory Animals of Southeast University, China (No. 20210302014; approval date: 2 March 2021). All testing was conducted by trained investigators who were blinded to the group allocations.

### 2.2. Diets

The SD and HFD used in this study were purchased from Trophic Animal Feed High-Tech Co., Ltd. (Nantong, China). The SD pellets (LAD3001G) provided 3.766 kcal/g energy, with 16.7% from fat, 64% from carbohydrates, and 19.3% from protein. The HFD pellets (TP23300) provided 5.0 kcal/g energy, with 60% from a lard and soybean oil mixture at a ratio of approximately 10:1, 20.6% from carbohydrates, and 19.4% from protein.

### 2.3. CUMS Protocol

In this study, we utilized a CUMS protocol based on previous methods [41,42]. The mice in the CUMS subgroups were subjected to two different unpredictable stressors every day, and the same stressor was not used on two consecutive days. The stresses included wet bedding for 24 h, bedding deprivation for 24 h, 45° cage tilting for 14 h, cage shaking for 30 min, physical restraint for 2 h, forced swimming at 8 °C for 1 min, and tail nipping (1 cm from the tip of the tail) for 1 min. The timeline for stressor exposure is given in Table 1.

### 2.4. Behavioral Tests

After four weeks of the specific dietary treatment covering almost the entire adolescence stage [39], all the mice were subjected to behavioral tests during the dark cycle (7 p.m. to 12 p.m.) in the following sequence: the sucrose preference test (SPT), the open field test (OFT), the elevated zero maze (EZM) test, and the forced swim test (FST). The experimental animals were transferred to the testing room 2 h prior to experimentation to adapt to the testing environment. Except the SPT, all behavioral assessments were recorded with a video camera and subsequently analyzed with a digital tracking system (Visutrack 3.0; Shanghai Xinruan Information Technology Company, Shanghai, China). The behavioral apparatus was meticulously cleaned with 70% ethanol and water between each mouse to avoid olfactory interference.

### 2.5. Open Field Test

The OFT was used to evaluate locomotor activity and levels of anxiety-like and depression-like behaviors in mice [43]. As previously described [44], the mice were individually placed in the corner of a plastic chamber (50 cm × 50 cm × 50 cm), and the behavior of each mouse was recorded for 5 min. During the analysis, the bottom of the plastic chamber was divided into a central square (25 cm × 25 cm) and a surrounding peripheral area. Both the total distance traveled in the area and the duration spent in the central area were measured.

### 2.6. Elevated Zero Maze Test

The EZM test was used to assess levels of anxiety-like [45] and depression-like [46] behaviors. The apparatus consisted of a white elevated zero maze with two open arms and two closed arms. Each mouse was placed in a closed arm, and their behavior was recorded for 5 min. Then, the duration spent in the open arms was measured.

### 2.7. Sucrose Preference Test

The SPT was performed to assess anhedonia, which is a core symptom of clinical depression [47]. Prior to the test, singly housed mice were presented with two identical bottles of tap water for 24 h to measure their basal water consumption. During the test, one of the two bottles was changed to contain a 1% sucrose solution for 24 h, and the positions of the bottles were switched after 12 h to account for potential location preference for drinking. Sucrose preference was then calculated as follows: sucrose preference (%) = [sucrose intake/(tap water intake + sucrose intake)] × 100%.

### 2.8. Forced Swim Test

The FST was conducted to evaluate behavioral despair, another primary symptom of depression [48]. The mice were individually placed in a transparent glass cylinder (13 cm in diameter and 18.5 cm deep) filled with 15 cm of fresh water (23–25 °C) for 6 min, and the immobility latency was measured. Immobility was defined as the minimum movement necessary to keep the animal’s nose above the water.

### 2.9. Detection of Serum Biochemical Parameters

After all the behavioral tests were completed, the mice were weighed and then deeply anesthetized with pentobarbital (100 mg/kg, intraperitoneally (i.p.)). Blood was harvested via cardiac puncture, allowed to clot at room temperature, and centrifuged at 4 °C to separate the serum, which was stored at −80 °C until analysis. To control for cyclic physiological variations, all female mice were euthanized for tissue collection specifically during the diestrus phase [49,50]. Upon completion of all the behavioral tests, the estrous cycle of female mice was monitored daily between 9 a.m. and 10 a.m. by vaginal lavage, following an established protocol [51]. The cycle stage was determined on the basis of the relative ratio of vaginal cell types: proestrus (predominantly nucleated epithelial cells), estrus (predominantly cornified epithelial cells), metestrus (predominantly both cornified epithelial cells and leukocytes), and diestrus (predominantly leukocytes) [49]. Representative vaginal cytology images for each estrous cycle stage are shown in Figure S1 (see Supplementary Information). The timing of tissue collection for female mice was staggered over a period from P61 to P64.

Serum levels of FFAs were determined using a nonesterified free fatty acid assay kit (A042-1-1; Nanjing Jiancheng Bioengineering Institute, Nanjing, China) [34]. Serum levels of leptin [34] (MOB00B; R&D Systems, Minneapolis, MN, USA), estradiol [52] (KGE014; R&D Systems), and testosterone [52] (KGE010; R&D Systems) were determined using the corresponding enzyme-linked immunosorbent assay (ELISA) kits following the manufacturer’s instructions.

### 2.10. Golgi Staining and Morphometric Analyses

For each subgroup, 3 randomly selected mice were used for Golgi staining. Unfixed brains were rapidly removed and immersed in equal volumes of solutions A and B from the FD Rapid Golgi Stain Kit (FD Neuro Technologies, Inc., Columbia, MD, USA) for 2 weeks in the dark, followed by incubation in solution C for 1 week [44]. The brains were coronally sectioned with a vibratome (Leica VT 1200S, Leica Biosystems) to a thickness of 150 µm. The brain sections were then stained with mixed working solution (solution D:solution E:double-distilled water  =  1:1:2), and images were acquired under a microscope (Olympus BX53, Tokyo, Japan). Morphological analysis of granule cells (DG region) and pyramidal cells (CA1 and CA3 regions) in both the dorsal hippocampus (dHP; bregma −0.94 to −2.30 mm) and vHP (bregma −2.46 to −3.80 mm) was performed using ImageJ software (version 1.52a; National Institutes of Health, Bethesda, MD, USA) [53,54].

For each animal, at least 7 neurons within each region of interest (ROI) meeting the following criteria were randomly selected and traced for Sholl analysis: (1) the neurons were relatively separate from the surrounding neurons and could be clearly identified; and (2) the neurons were well stained without obvious disruption [55]. Sholl analysis was performed to determine the total dendritic length, number of dendritic branches, number of dendritic intersections with concentric rings with increasing radii of 10 µm, and the area under the curve (AUC) of the Sholl intersection.

The spine densities of secondary branches at least 15 µm in length located approximately 10 µm from the bifurcation point are reported as the number of spines per 10 µm of dendrite length. At least 6 well-stained neurons within each ROI from each animal were randomly selected for dendritic spine density analysis.

### 2.11. Immunofluorescence Staining

For each subgroup, 5 randomly selected mice were used for immunofluorescence staining. The mice were perfused with 0.9% saline for 3 min and 4% paraformaldehyde (PFA) for 4 min. The brains were then rapidly excised, fixed with 4% PFA for 8 h, cryoprotected in 30% sucrose until they sank to the bottom of the container, embedded in optimal cutting temperature compound, and stored at −80 °C. In accordance with the mouse brain atlas [53], serial coronal sections (40 µm thick) of the dHP (bregma −0.94 to −2.30 mm) and vHP (bregma −2.46 to −3.80 mm) were obtained using a freezing microtome (Leica Cryostat Microtome 1900, Leica Biosystems, Deer Park, IL, USA) [54]. For each analysis, two to three coronal sections at 160 µm intervals were selected from each mouse for immunohistochemical staining. After being blocked with blocking solution (5% normal donkey serum and 0.3% Triton-X-100 in 0.1 M phosphate-buffered saline (PBS)), the sections were incubated with the following primary antibodies overnight at 4 °C: rabbit anti-ionized calcium-binding adaptor molecule 1 (Iba1; 019-19741, 1:1000; Wako, Osaka, Japan), goat anti-Iba1 (011-27991, 1:600; Wako), rat anti-cluster of differentiation 68 (CD68; MCA1957, 1:1000; Bio-Rad, Oxford, UK), rabbit anti-postsynaptic density 95 (PSD95; ab18258, 1:1000; Abcam, Cambridge, UK), mouse anti-tumor necrosis factor-alpha (TNF-α; sc-52746, 1:100; Santa Cruz, CA, USA), and mouse anti-androgen receptor (AR; sc-7305, 1:50; Santa Cruz, CA, USA). After being washed with PBS, the sections were incubated with the appropriate secondary antibody for 2 h at room temperature in the dark. Finally, the cell nuclei were stained with 4,6-diamidino-2-phenylindole (DAPI; C1027, 1:600; Beyotime, Shanghai, China). Negative controls included sections incubated without primary or secondary antibodies.

### 2.12. Isolation and Culture of Primary Hippocampal Microglia

A total of 124 mice (12 males and 112 females) were used to isolate primary hippocampal microglia across 17 independent isolations, 14 of which were used for immunofluorescence staining, and 3 of which were used for qRT–PCR. Primary hippocampal microglia were isolated from mice as previously described [56,57]. Briefly, male and female C57BL/6J mice (8 weeks of age) were obtained from Hangzhou Ziyuan Experimental Animal Technology Co., Ltd. (Hangzhou, China; SCXK(ZHE)2024-0004) and acclimated until P61, which corresponded to the in vivo experimental endpoint. Following deep anesthesia, the hippocampal tissues were quickly excised. The hippocampal tissue was dissociated into single-cell suspensions using a high-activity brain enzymatic digestion kit (DHABE-5003, RWD, Shenzhen, China) and a single-cell suspension dissociator (DSC-200, RWD) following the manufacturer’s instructions. After the removal of cell debris using a High Efficiency Debris Removal Kit (DHDR-5006, RWD), the cells were resuspended in magnetic activated cell sorting (MACS) buffer (PBS with 2 mM EDTA and 0.5% BSA) and then incubated with anti-CD11b microbeads (130-126-725, Miltenyi Biotec, Bergisch Gladbach, Germany). Microglia were isolated through magnetic cell sorting using an LS column (130-042-201; Miltenyi Biotec).

Primary microglia were maintained in a humidified atmosphere (5% CO_2_, 95% air) at 37 °C for 2 to 4 days in culture medium (DMEM/F12 supplemented with 10% fetal bovine serum (FBS) and 1% penicillin/streptomycin) and harvested for subsequent experiments.

Primary microglia were approximately 97.93% pure, as determined by staining for Iba1 (019-19741, Wako) (Figure S2). Cell viability was determined by using a calcein-AM/PI live/dead viability assay kit (C2015S; Beyotime). Calcein-AM^+^ (green) cells were considered alive, while propidium iodide (PI)^+^ (red) cells were considered dead [58]. Cell viability is expressed as the ratio of the number of calcein-AM^+^ cells to the total number of calcein-AM^+^ plus PI^+^ cells [59]. Primary microglial viability was approximately 97.37% (Figure S3). For each independent experiment, five random fields per culture were imaged. All cells within the entire imaging area were analyzed by trained investigators using the Cell Counter plugin in ImageJ software (1.52a).

### 2.13. In Vitro Phagocytosis Assay and Immunofluorescence Staining

For immunofluorescence staining, primary microglia were seeded at a density of 2.5 × 10^5^ cells per well. The serum was thawed at room temperature and diluted to 5% in culture medium [8] before being applied to the cells without further processing. Following serum starvation for 1 h, primary hippocampal microglia obtained from male and female mice were treated with 5% serum from age- and sex-matched SD- or HFD-fed mice for 12 h. After treatment, the cells were washed with PBS twice and then treated with culture medium containing pHrodo Red Zymosan Bioparticles (P35364, 1:4000; Thermo Fisher Scientific, Waltham, MA, USA) for 1 h in the dark. Afterward, the cells were fixed in 4% PFA, washed, blocked, incubated with primary antibodies (anti-Iba1 antibody, 019-19741; Wako; and anti-AR antibody, sc-7305; Santa Cruz, CA, USA), and then incubated with secondary antibodies.

In a separate experiment, primary hippocampal microglia obtained from female mice were treated with 5% serum from age-matched female SD- or HFD-fed mice in combination with enzalutamide (MDV3100, an AR antagonist; 10 μM; Selleck Chemicals, Houston, TX, USA; Enz) [60,61] or vehicle control (0.1% DMSO; Veh). These cells were then subjected to the same phagocytosis assay described above and immunostained with primary antibodies against Iba1, AR, and TNF-α (sc-52746; Santa Cruz, CA, USA), followed by treatment with secondary antibodies.

### 2.14. Immunofluorescence Image Acquisition and Analysis

Images were captured using a confocal microscope (FV3000; Olympus, Tokyo, Japan) with 10×, 20× and 40× objectives. The confocal image stacks were obtained at a resolution of 1024 × 1024 pixels with a step size of 1 µm (for brain section analysis) or 0.5 µm (for primary microglia analysis). The brain ROIs (DG molecular layer, CA3 stratum radiatum, and CA1 stratum radiatum of both the dorsal and ventral hippocampus) were determined according to the mouse brain atlas (Figure S4) [53]. At least ten Iba1^+^ cells with clearly visible cell bodies within each ROI from each animal were randomly analyzed by a trained investigator who was blinded to the experimental conditions using ImageJ software (1.52a) or Imaris software (9.8.0; Bitplane, Oxford, UK).

The microglial density, territory area (defined as the two-dimensional area outlined by the outermost points of the dendritic processes of an Iba1^+^ cell), soma area, morphological index (ratio of soma to territory area of the Iba1^+^ cells [62]), and CD68 expression were analyzed using ImageJ software to assess microglial status [44]. CD68 expression was scored according to a previously described protocol. A score of zero denoted the absence of CD68^+^ signals or signals with a diameter of less than 1 µm; a score of one signified the presence of at least one punctate signal with a diameter greater than 1 µm and signals covering less than one-third of the Iba1^+^ cell soma area; a score of two signified signals covering between one-third and two-thirds of the soma area; and a score of three indicated signals covering more than two-thirds of the soma area [44].

To quantitatively assess microglial PSD95^+^ inclusions, the three-dimensional surfaces of the Iba1^+^ cells were reconstructed by using the “surface creation” wizard of Imaris software as described previously [8]. PSD95^+^ signals within Iba1^+^ cells were extracted by using the mask function of Imaris, after which the surfaces were created and analyzed. The volume of PSD95^+^ puncta within Iba1^+^ cells was quantified and subsequently analyzed.

To quantify the TNF-α^+^ and zymosan^+^ signals inside microglia, three-dimensional surfaces of the Iba1^+^ cells were reconstructed using Imaris software as described above. The integrated density (IntDen) values of the TNF-α^+^ and zymosan^+^ signals within Iba1^+^ cells were quantified and analyzed.

To quantify AR nuclear localization, the three-dimensional surfaces of the Iba1^+^ cells were reconstructed using Imaris software as described above, and a new channel for the microglial nucleus was subsequently created by using the mask function to extract DAPI^+^ signals within Iba1^+^ cells. The IntDen values of the AR^+^ signals within both the nuclei of Iba1^+^ cells (referred to as nuclear AR IntDen) and whole Iba1^+^ cells (referred to as cellular AR IntDen) were quantified. AR nuclear localization in microglia was subsequently quantified as the percentage of nuclear to cellular AR IntDen [63,64].

### 2.15. Quantitative Real-Time PCR (qRT–PCR)

For qRT–PCR, primary microglia were seeded at a density of 3.5 × 10^5^ cells per well. Primary hippocampal microglia obtained from female mice were treated with 5% serum from age-matched female SD- or HFD-fed mice in combination with Enz or Veh as described above. After the cells were washed with PBS, total RNA was extracted using an RNA-Quick Purification Kit (ES-RN001; Yishan, Shanghai, China) and reverse-transcribed to cDNA with a Reverse Transcription Kit (R212-02; Vazyme, Nanjing, China). Quantitative RT–PCR reactions were performed with Taq Pro Universal SYBR qPCR Master Mix (Q712-02; Vazyme) according to the manufacturer’s instructions. The primer sequences are shown in Table 2. The relative expression of the *TNF-α* gene was normalized to that of the housekeeping gene *β-actin* using the 2^−ΔΔCt^ method. 

### 2.16. Statistical Analysis

GraphPad Prism 9 (GraphPad Software Inc., San Diego, CA, USA) or Origin software (Version 2022, Origin Lab Corp., Northampton, MA, USA) was used for statistical analysis of the data and graph construction. The normality of the data was examined with the Kolmogorov–Smirnov test or Shapiro–Wilk test, and nonnormally distributed data were subjected to log transformation and tested again for normality. For sex-stratified analyses, comparisons between experimental groups of the same sex were performed using two-tailed unpaired Student’s *t* test or two-way analysis of variance (ANOVA) as appropriate, following confirmation of a normal distribution. In the case of significant interaction effects, Tukey’s post hoc test was performed. If the data were not normally distributed after transformation, comparisons of the data between experimental groups of the same sex were performed using the Mann–Whitney test or the Kruskal–Wallis test followed by Dunn’s test. Only comparisons that demonstrated significant differences that are relevant to our hypotheses are reported in the figures. For in vitro experiments, the sample size indicates the number of independent experiments. The values are presented as the means ± standard errors of the means (SEMs), and differences were considered statistically significant at *p* < 0.05. The detailed two-way ANOVA results are shown in Tables S1–S8 (see Supplementary Information). All the investigators were blinded to the group allocations during data collection and analysis.

Radar charts (generated by Origin software) were used to display the relationships among different parameters simultaneously [65]. The values were normalized by dividing each data point by the average of the sex-matched SD-Ctrl group.

Correlations between parameters were assessed using Pearson’s correlation analysis.

## 3. Results

### 3.1. Sex-Stratified Characterization of Behavioral and Circulating Humoral Responses to Adolescent HFD Consumption in Mice

**Figure 1:**
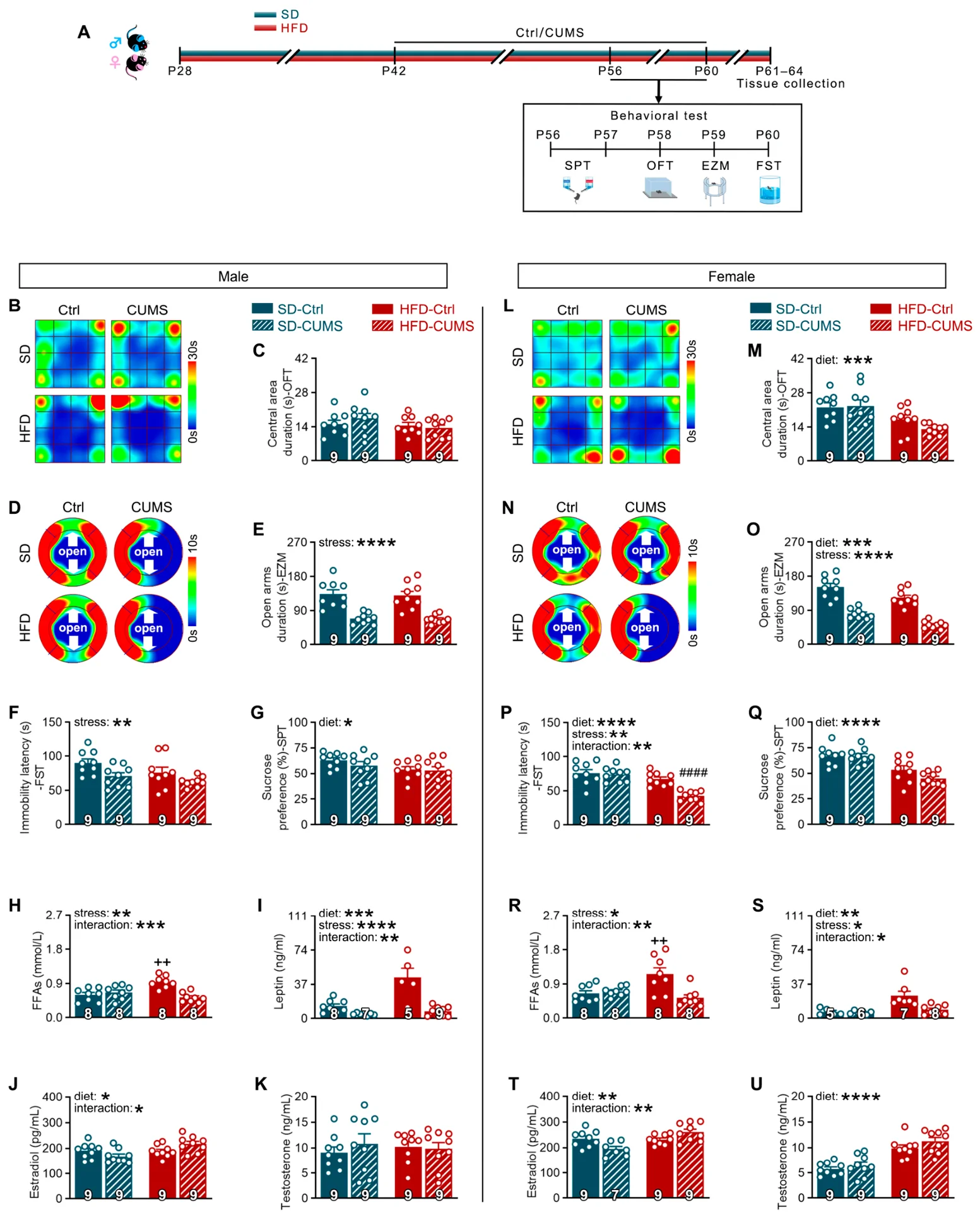
Sex-stratified characterization of behavioral and circulating humoral responses to adolescent HFD consumption in mice. (**A**) Schematic of the experimental design and behavioral tests. (**B**,**D**,**L**,**N**) Representative trajectory heatmaps in the OFT (**B**,**L**) and EZM test (**D**,**N**) for male and female mice. (**C**,**M**) Central area duration in the OFT for male and female mice. (**E**,**O**) Open arms duration in the EZM test for male and female mice. (**F**,**P**) Immobility latency in the FST for male and female mice. (**G**,**Q**) Sucrose preference during the SPT for male and female mice. (**H**–**K**), and (**R**–**U**) Serum levels of FFAs (**H**,**R**), leptin (**I**,**S**), estradiol (**J**,**T**), and testosterone (**K**,**U**) in male and female mice. The data are shown as the means ± SEMs. *n* = 5–9 mice per group, with exact *n* values indicated at the base of each bar. The data were analyzed separately for each sex by two-way ANOVA with diet and stress as factors, followed by Tukey’s post hoc test where appropriate. Significant main effects of diet or stress and diet × stress interactions are indicated above the corresponding panels as “diet”, “stress”, or “interaction”, respectively; only statistically significant effects are shown. * *p <* 0.05, ** *p <* 0.01, *** *p <* 0.001, and **** *p <* 0.0001 indicate the significance level of the corresponding main effect or interaction. ++ *p <* 0.01 vs. the sex-matched SD-Ctrl group; #### *p <* 0.0001 vs. the sex-matched SD-CUMS group.

A battery of behavioral tests was performed to assess the effects of adolescent HFD consumption on emotional behavior and stress responsiveness in male and female mice (Figure 1A). Locomotor activity was unaffected by either diet or CUMS (males: Figure S5A; females: Figure S5C), excluding gross activity changes as a confounding factor.

As expected, CUMS induced depression-related behaviors in both sexes, as evidenced by less time spent in the open arms of the EZM (males: Figure 1D,E; females: Figure 1N,O) and decreased immobility latency in the FST (males: Figure 1F; females: Figure 1P). With respect to the effects of the HFD, females showed significant reductions in central area duration in the OFT (Figure 1L,M), open arm time in the EZM (Figure 1N,O), and immobility latency in the FST (Figure 1P), whereas these measures were not significantly altered in males (Figure 1B–F). In contrast, sucrose preference decreased after consumption of a HFD in both sexes (males: Figure 1G; females: Figure 1Q), with no changes in total fluid intake (males: Figure S5B; females: Figure S5D). These findings indicate that a HFD induced anhedonia-like behavior in both sexes, whereas other emotional behavioral changes were observed only in females. Notably, a significant diet × stress interaction was observed for immobility latency in the FST in females but not in males (Figure 1P). Post hoc analysis revealed that immobility latency was further reduced in HFD-CUMS females compared with that in SD-CUMS females, suggesting that adolescent HFD consumption amplifies the behavioral response to CUMS in females.

Adolescent HFD consumption also altered the circulating metabolic and endocrine profiles. In both sexes, CUMS significantly decreased serum FFAs levels (males: Figure 1H; females: Figure 1R). Although no main effect of diet was detected, a significant diet × stress interaction was observed in both sexes, with post hoc analyses showing elevated FFAs levels in HFD-Ctrl mice compared with those in SD-Ctrl mice. Serum leptin levels were increased by HFD consumption and decreased by CUMS in both sexes (males: Figure 1I; females: Figure 1S), indicating opposing effects of diet and stress. Serum estradiol levels were elevated by HFD consumption in both sexes; however, CUMS did not significantly affect serum estradiol levels (males: Figure 1J; females: Figure 1T). Notably, the serum testosterone levels were elevated in HFD-fed females (Figure 1U) but not males (Figure 1K) and was not significantly affected by CUMS.

Collectively, these sex-stratified analyses indicate that adolescent HFD consumption induces anhedonia-like behavior in both sexes, with broader behavioral impairments and greater behavioral responses to stress observed only in females. This phenotype is accompanied by elevated levels of circulating testosterone only in females, suggesting a possible association between HFD-related endocrine alterations and behavioral outcomes.

### 3.2. Sex-Stratified Characterization of Hippocampal Neurostructural Responses to Adolescent HFD Consumption in Mice

**Figure 2:**
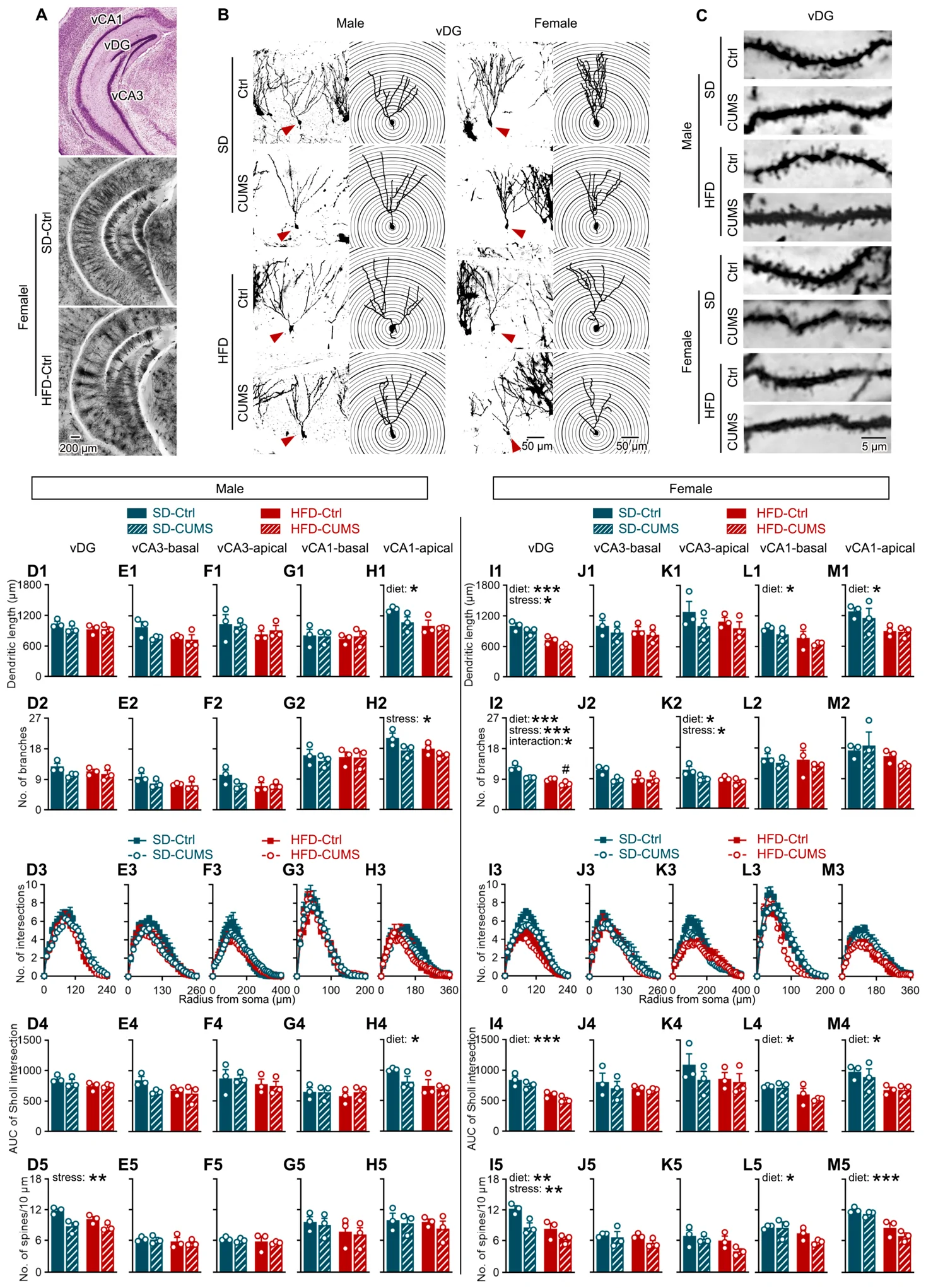
Sex-stratified characterization of ventral hippocampal neurostructural responses to adolescent HFD consumption in mice. (**A**) Top panel: regions of interest (ROIs) in the ventral hippocampus in the mouse brain atlas [53]. Representative images of Golgi-stained cells in the ventral hippocampus of female SD-Ctrl (**middle panel**) and HFD-Ctrl (**bottom panel**) mice. (**B**) Representative Golgi-stained granule cells in the vDG region (indicated by red arrowheads, left) and their traces along with a graphic description of the Sholl analysis parameters (right). (**C**) Representative photomicrographs of the secondary branches of granule cells in the vDG region. (**D**–**M**) Dendritic lengths (**D1**–**M1**), numbers of dendritic branches (**D2**–**M2**), numbers of dendritic intersections with the equidistant Sholl spheres (**D3**–**M3**), AUCs of the Sholl intersection (**D4**–**M4**), and numbers of dendritic spines/10 µm (**D5**–**M5**) in the ROIs of male and female mice. The data are shown as the means ± SEMs. *n* = 3 mice per group. The data were analyzed separately for each sex by two-way ANOVA with diet and stress as factors, followed by Tukey’s post hoc test where appropriate. Significant main effects of diet or stress and diet × stress interactions are indicated above the corresponding panels as “diet”, “stress”, or “interaction”, respectively; only statistically significant effects are shown. * *p <* 0.05, ** *p <* 0.01, and *** *p <* 0.001 indicate the significance level of the corresponding main effect or interaction. # *p <* 0.05 vs. the sex-matched SD-CUMS group.

We next examined whether the behavioral phenotype was accompanied by changes in the structure of the hippocampus along the dorsal–ventral axis. Dendritic complexity, indexed by total dendritic length, branch number, and Sholl analysis (area under the curve, AUC), as well as dendritic spine density were quantified in the granule cells of the DG and pyramidal neurons of the CA1 and CA3 subregions in both the dHP (Figure S6) and vHP (Figure 2).

In the dHP, adolescent HFD consumption altered the neurostructure only in females, as shown by significantly reduced dendritic complexity and spine density in the dorsal DG (dDG) of females (Figure S6A–C,I), whereas these parameters were not significantly affected in males (Figure S6D–H). No significant main effects of CUMS on dorsal hippocampal neurostructure were detected in either sex (Figure S6D–M), suggesting limited stress sensitivity in this region under the experimental conditions.

In contrast, more extensive alterations were observed in the vHP. In females, HFD consumption significantly reduced dendritic complexity across multiple subregions, including the ventral DG (vDG), CA3 (vCA3), and CA1 (vCA1), and decreased spine density in the vDG and vCA1 (Figure 2A–C,I,K–M). In males, the HFD only significantly decreased dendritic complexity in the vCA1 (Figure 2H). These findings indicate that ventral hippocampal circuits are vulnerable to adolescent HFD consumption in both sexes, with more extensive neurostructural changes in females.

CUMS significantly affected ventral hippocampal structure, including the vDG and vCA3 of females and the vDG and vCA1 of males (Figure 2B–D,H,I,K). Importantly, a significant diet × stress interaction was detected for the number of dendritic branches in the vDG of females but not males (Figure 2(I2)). Post hoc analyses revealed a greater reduction in branch number in HFD-CUMS females than in SD-CUMS females, indicating that adolescent HFD consumption amplifies stress-related neurostructural impairments in females.

Collectively, these sex-stratified analyses indicate that adolescent HFD consumption is associated with hippocampal neurostructural alterations in both sexes, with the effects predominantly detected in the ventral hippocampus with greater vulnerability among females.

### 3.3. Sex-Stratified Characterization of Hippocampal Microglial Responses to Adolescent HFD Consumption in Mice

**Figure 3:**
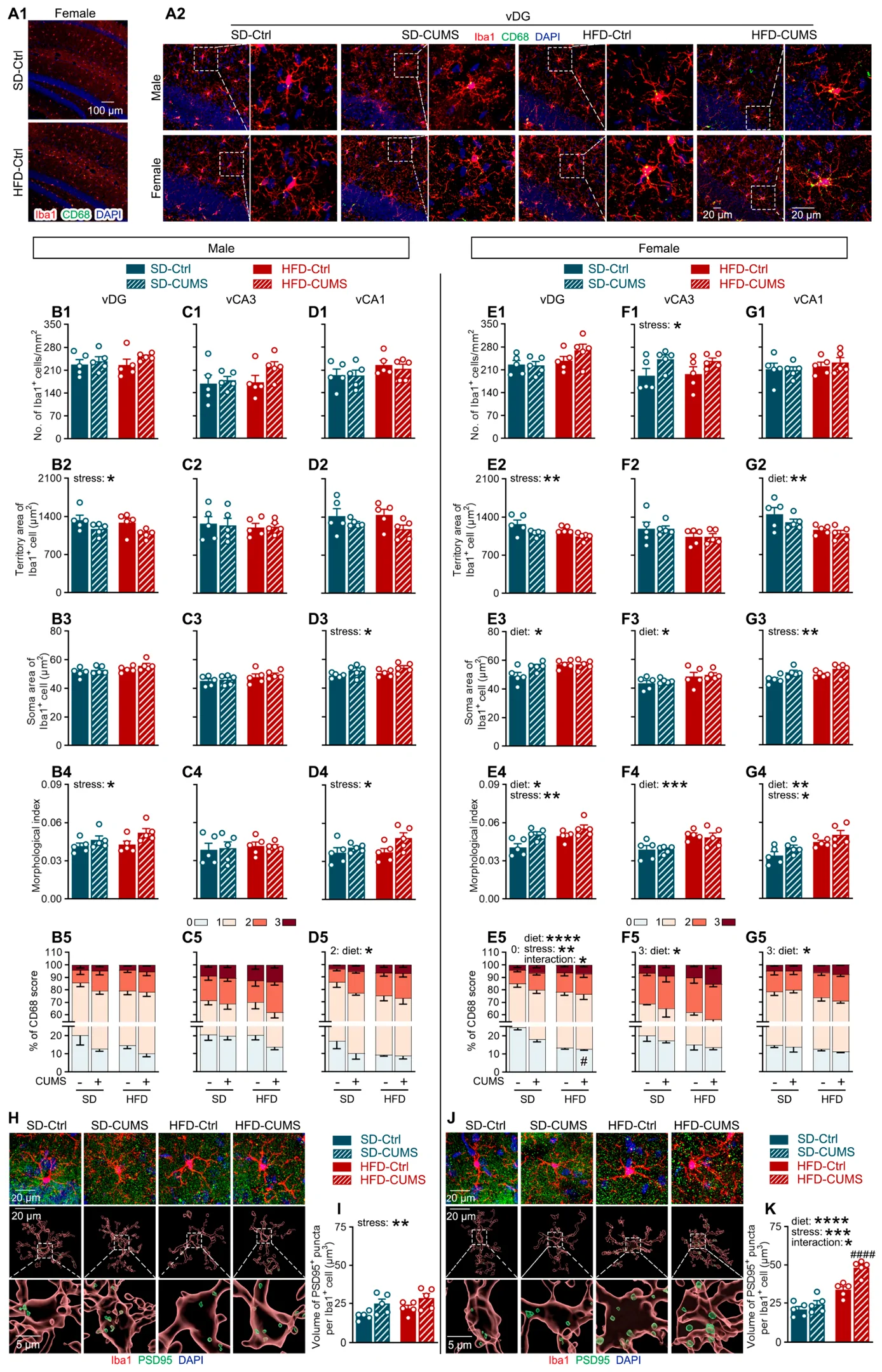
Sex-stratified characterization of ventral hippocampal microglial responses to adolescent HFD consumption in mice. (**A1**) Representative low-magnification images of immunofluorescence staining for Iba1 (red), CD68 (green), and DAPI (blue) in the ventral hippocampi of female SD-Ctrl and HFD-Ctrl mice (10× magnification). (**A2**) Representative high-magnification images of immunofluorescence staining for Iba1 (red), CD68 (green), and DAPI (blue) in the vDG regions of all groups (40× magnification). Areas indicated by dashed lines are shown at higher magnification on the right. (**B**–**G**) Microglial density (**B1**–**G1**), territory area (**B2**–**G2**), soma size (**B3**–**G3**), morphological index (**B4**–**G4**), and percentage of microglia with CD68^+^ scores ranging from 0 to 3 (**B5**–**G5**) in the ROIs of male and female mice. (**H**,**J**) Representative images of immunofluorescence staining for Iba1 (red), PSD95 (green), and DAPI (blue) and three-dimensional reconstructions of the microglia (red) and PSD95 puncta (green) inside microglia in the vDG region of male and female mice. Areas indicated by dashed lines are shown at higher magnification below. (**I**,**K**) Volume of PSD95 puncta per Iba1^+^ cell in the vDG region of male and female mice. − and + indicate the Ctrl and CUMS subgroups, respectively. The data are shown as the means ± SEMs. *n* = 5 mice per group. The data were analyzed separately for each sex by two-way ANOVA with diet and stress as factors, followed by Tukey’s post hoc test where appropriate. Significant main effects of diet or stress and diet × stress interactions are indicated above the corresponding panels as “diet”, “stress”, or “interaction”, respectively; only statistically significant effects are shown. * *p <* 0.05, ** *p <* 0.01, *** *p <* 0.001, and **** *p <* 0.0001 indicate the significance level of the corresponding main effect or interaction. # *p <* 0.05, #### *p <* 0.0001 vs. the sex-matched SD-CUMS group.

Given the prominent structural alterations in the ventral hippocampus, we next assessed microglial status in the same hippocampal axis. We quantified microglial density, territory area, soma size, morphological index, and CD68 expression in the DG molecular layer, CA3 stratum radiatum, and CA1 stratum radiatum of both the dHP (Figure S7) and vHP (Figure 3) using immunostaining for Iba1 (a microglial marker) and CD68 (a marker of phagocytic activity).

In the dHP, adolescent HFD consumption was associated with increased soma size and elevated CD68 expression in the dDG of females (Figure S7A,E), whereas these parameters were not significantly altered in males (Figure S7B–D). No significant main effects of CUMS were detected in either sex (Figure S7B–G), indicating limited stress responsiveness of dorsal hippocampal microglia, which is consistent with the relatively mild neurological structural changes observed in this region.

In contrast, more robust alterations were detected in the vHP. In females, HFD consumption was associated with widespread microglial activation-like changes, including a reduced territory area (vCA1), increased soma size (vDG and vCA3), an elevated morphological index (vDG, vCA3, and vCA1), and increased CD68 expression across subregions (Figure 3A,E–G). In males, HFD consumption was associated with limited microglial activation-like changes, with increased CD68 expression in the vCA1 (Figure 3D). These findings indicate that ventral hippocampal microglia are sensitive to adolescent HFD consumption in both sexes, with a broader response in females.

CUMS further modulated microglial status in the vHP, with significant effects observed in females across all three subregions, whereas in males, the effects were limited to the vDG and vCA1 (Figure 3B,D–G). Importantly, a significant diet × stress interaction was detected for CD68 expression in the vDG of females but not males (Figure 3(E5)). Post hoc analysis revealed a reduction in the proportion of CD68-low microglia in HFD-CUMS females compared with that in SD-CUMS females, which was consistent with the increase in microglial phagocytic activity under combined HFD and stress conditions.

To determine whether these microglial changes were associated with altered synaptic remodeling, we quantified microglial synaptic inclusions by coimmunostaining for Iba1 and PSD95 (a postsynaptic protein) in the DG molecular layer of the vHP, where the most prominent structural deficits were observed. The volume of microglial PSD95^+^ inclusions significantly increased in females (Figure 3J,K) but not males (Figure 3H,I) following HFD exposure, indicating increased microglia-mediated synaptic engulfment in females. CUMS increased the volume of microglial PSD95^+^ inclusions in both sexes (Figure 3H–K), although a significant diet × stress interaction was detected in females only (Figure 3K), with post hoc analyses showing greater inclusion volume in HFD-CUMS females than in SD-CUMS females. These results link adolescent HFD consumption to a microglial phagocytic phenotype in females that parallels ventral hippocampal structural deficits.

To integrate behavioral, structural, and molecular alterations, radar plots were constructed to simultaneously visualize serum testosterone levels, microglial parameters, synaptic inclusions, and depression-related behaviors. Distinct contour patterns were observed between the HFD and SD groups, with more pronounced divergence in females (Figure S8A,B). Notably, HFD-CUMS females exhibited the most extreme profile, characterized by elevated serum testosterone levels, increased microglial soma size and synaptic inclusions, and reduced immobility latency in the FST. These integrated profiles highlight coordinated alterations across endocrine, cellular, and behavioral domains, and suggest that HFD consumption amplifies stress effects predominantly in females.

### 3.4. Sex-Stratified Characterization of Hippocampal Microglial AR Nuclear Localization and TNF-α Expression Responses to Adolescent HFD Consumption in Mice

The behavioral and microglial alterations induced by adolescent HFD consumption in females were accompanied by elevated circulating testosterone levels. Given that testosterone readily crosses the blood–brain barrier [66] and exerts cellular effects via the AR [67] and that microglia express functional AR [38], we next examined whether androgen signaling is associated with the observed changes in microglia in the hippocampus.

We assessed AR nuclear localization—a key indicator of receptor activation [68,69,70]—in hippocampal microglia within the DG molecular layer of the vHP, which is the same region that exhibited pronounced microglial synaptic inclusions. Because serum testosterone levels were altered by a HFD in females but not males and were not significantly affected by CUMS, analyses in this section were restricted to nonstressed Ctrl animals.

**Figure 4:**
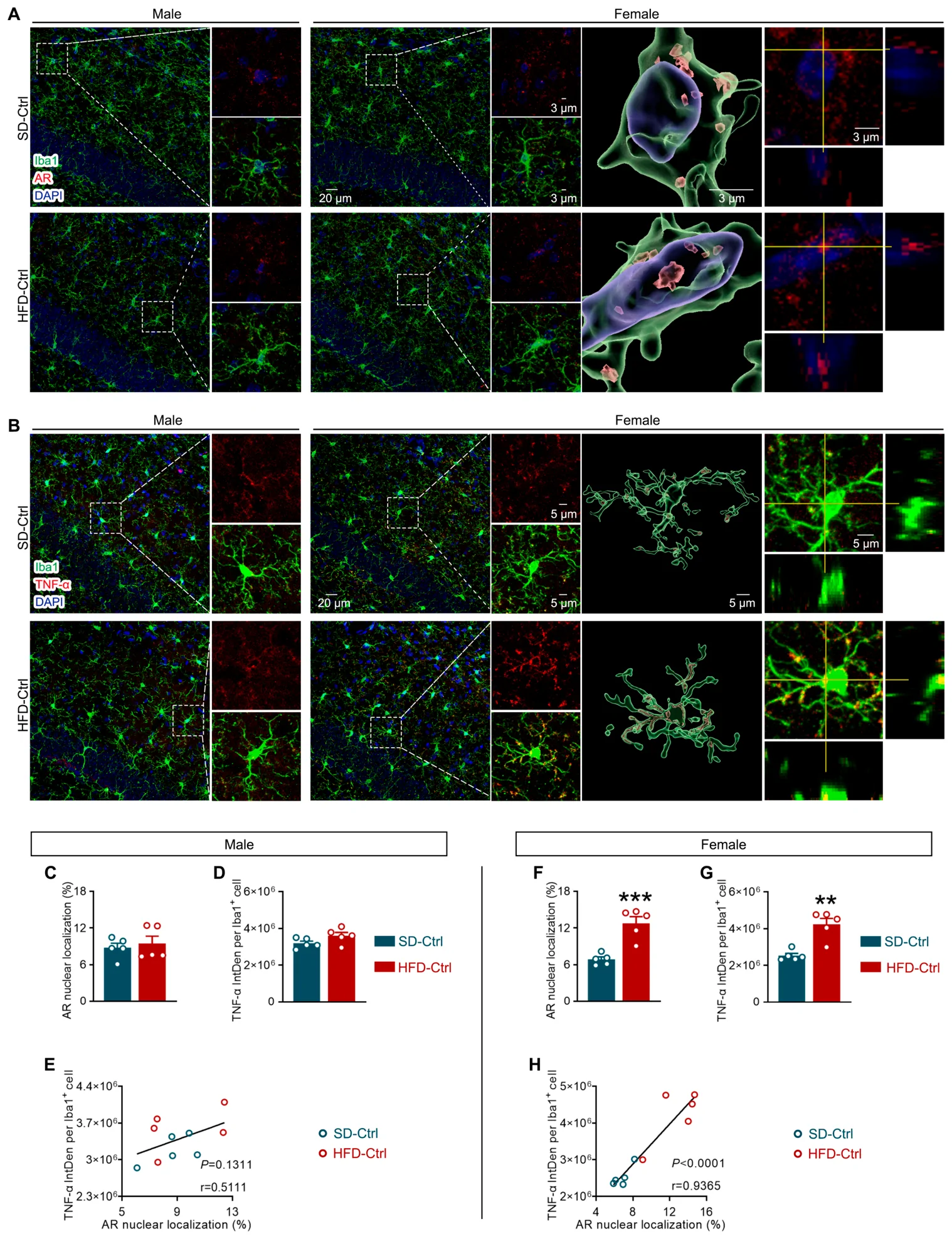
Sex-stratified characterization of hippocampal microglial AR nuclear localization and TNF-α expression responses to adolescent HFD consumption in mice. (**A**) Representative images of immunofluorescence staining for Iba1 (green), AR (red), and DAPI (blue) in the vDG regions of male and female SD-Ctrl and HFD-Ctrl mice. Areas indicated by dashed lines are shown at higher magnification and as split channels. Three-dimensional reconstructions of the soma regions of the indicated microglia (green) and the AR (red) inside the microglia from female SD-Ctrl and HFD-Ctrl mice. High-magnification images with orthogonal views showing AR^+^ signals (red) within microglial nuclei (blue) are presented. Yellow lines indicate the orthogonal section planes used to generate the side views. (**B**) Representative images of immunofluorescence staining for Iba1 (green), TNF-α (red), and DAPI (blue) in the vDG region of male and female SD-Ctrl and HFD-Ctrl mice. Areas indicated by dashed lines are shown at higher magnification and as split channels. Three-dimensional reconstructions of microglia (green) and TNF-α (red) inside the microglia in the indicated areas from female SD-Ctrl and HFD-Ctrl mice. High-magnification images with orthogonal views showing TNF-α^+^ signals (red) within microglia (green) are presented. Yellow lines indicate the orthogonal section planes used to generate the side views. (**C**,**D**,**F**,**G**) AR nuclear localization (**C**,**F**) and the integrated density (IntDen) of TNF-α per Iba1^+^ cell (**D**,**G**) in the vDG regions of male and female SD-Ctrl and HFD-Ctrl mice. (**E**,**H**) Linear regression analysis of AR nuclear localization and the IntDen of TNF-α per Iba1^+^ cell in male and female mice. The data are shown as the means ± SEMs. *n* = 5 mice per group. The data were analyzed separately for each sex by Student’s *t* test. ** *p <* 0.01, *** *p <* 0.001 vs. the sex-matched SD-Ctrl group (Student’s *t* test).

Consistent with previous reports [38], AR immunoreactivity was detected in Iba1^+^ microglia (Figure 4A). Notably, HFD consumption significantly increased AR nuclear localization in microglia from female (Figure 4F, *p* = 0.0009) but not male (Figure 4C) mice, indicating enhanced androgen signaling in females. To further evaluate downstream signaling, we examined the expression of TNF-α, a cytokine previously reported to be upregulated following AR activation and commonly associated with activated microglial states [71,72]. Consistent with the AR activation patterns, TNF-α expression in hippocampal microglia was significantly elevated in females (Figure 4B,G, *p* = 0.0014) but not in males (Figure 4D) following HFD exposure.

Correlation analysis revealed a strong positive correlation between microglial AR nuclear localization and TNF-α expression in females (Figure 4H, *p* < 0.0001, r = 0.9365), whereas no significant relationship was detected in males (Figure 4E). These findings suggest a coordinated activation of androgen signaling and proinflammatory pathways in female microglia in response to adolescent HFD consumption. To integrate endocrine, cellular, and behavioral alterations, radar plots were generated to visualize serum testosterone levels, microglial androgen signaling (AR nuclear localization and TNF-α expression), microglial parameters, and depression-related behaviors. Distinct contour profiles were observed between the HFD and SD groups, with more pronounced divergence among females (Figure S9A,B). Notably, HFD-fed females exhibited a coordinated phenotype characterized by elevated testosterone levels, increased AR activation and TNF-α expression, increased microglial soma size and synaptic inclusions, and reduced immobility latency in the FST.

Collectively, these findings support the coordinated activation of androgen-responsive and inflammatory microglial signaling in females following adolescent HFD exposure.

### 3.5. Effects of Circulating Factors from Adolescent HFD-Fed Mice on Microglial AR and Phagocytic Responses In Vitro

**Figure 5:**
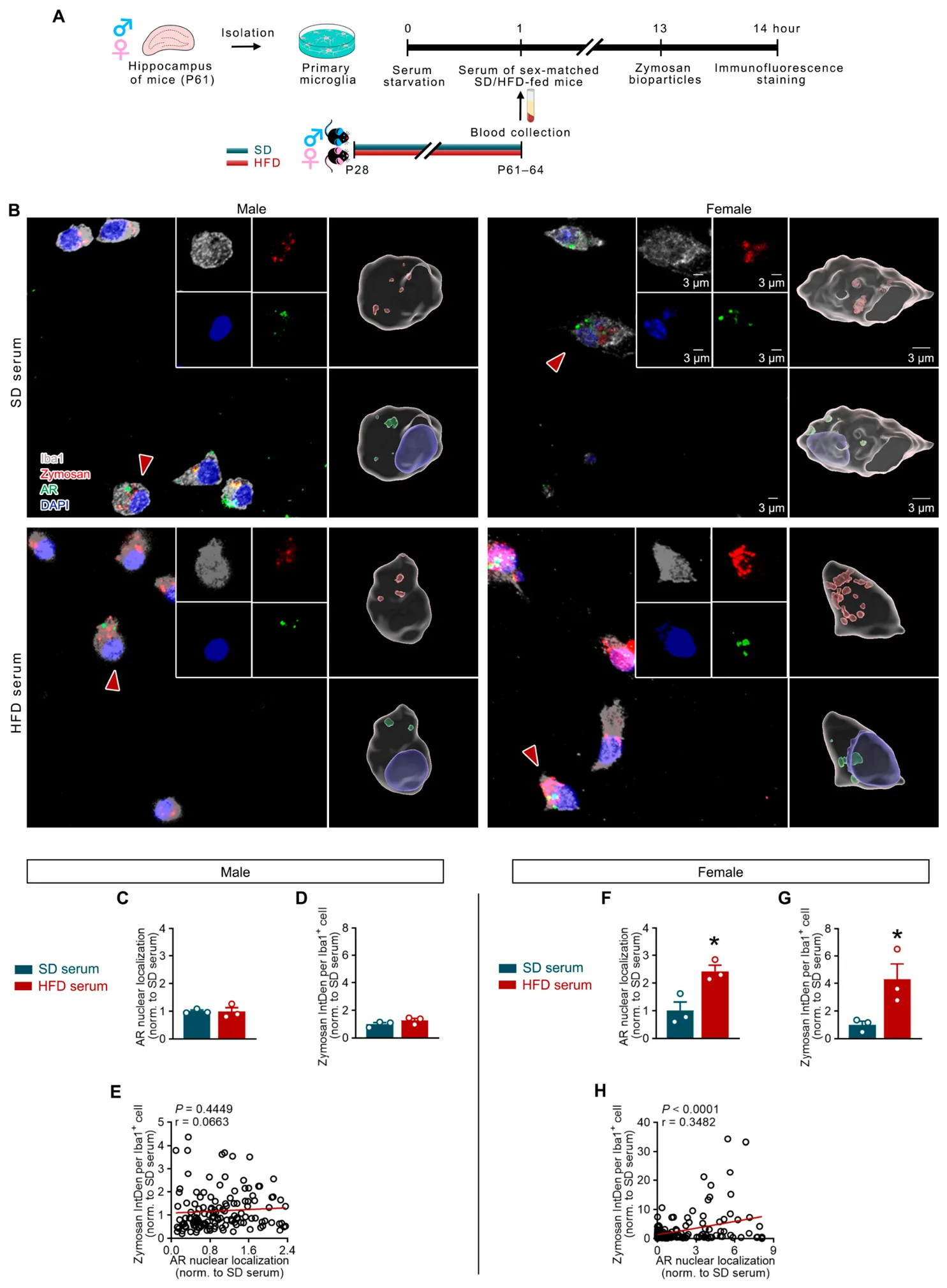
Effects of circulating factors from adolescent HFD-fed mice on microglial AR and phagocytic responses in vitro. (**A**) Design of the in vitro experiment in which primary hippocampal microglia from male and female mice were treated with serum from age- and sex-matched SD- or HFD-fed mice. (**B**) Representative images of immunofluorescence staining for Iba1 (gray), zymosan (red), AR (green) and DAPI (blue) in male and female primary microglia. High-magnification images showing the different fluorescence channels and three-dimensional reconstructions of microglia (gray), zymosan (red), AR (green) and DAPI (blue), as indicated by the arrowheads, are presented. (**C**,**D**,**F**,**G**) AR nuclear localization (**C**,**F**) and the IntDen of zymosan per Iba1^+^ cell (**D**,**G**) in male and female primary microglia. (**E**,**H**) Linear regression analysis of AR nuclear localization and the IntDen of zymosan per Iba1^+^ cell in male and female primary microglia. The data were normalized by dividing each data point by the average of the sex-matched SD serum group. The data are shown as the means ± SEMs. In (**C**,**D**,**F**,**G**), *n* = 3 independent experiments. The data were analyzed separately for each sex by Student’s *t* test. * *p <* 0.05 vs. the sex-matched SD serum group (Student’s *t* test).

To determine whether circulating factors drive the microglial responses observed in vivo, primary hippocampal microglia from male and female mice were treated with serum from sex-matched HFD- or SD-fed mice (Figure 5A).

Serum from HFD-fed females significantly increased both AR nuclear localization (Figure 5B,F, *p* = 0.0198) and phagocytic activity (Figure 5B,G, *p* = 0.0462) in female-derived microglia compared with serum from SD-fed controls. No significant changes were detected in male-derived microglia treated with serum from HFD-fed males (Figure 5B–D). These results indicate that female-derived microglia are responsive to circulating factors. Correlation analysis revealed a positive correlation between AR nuclear localization and phagocytic activity in female microglia (Figure 5H, *p* < 0.0001, r = 0.3482), whereas no significant relationship was detected in males (Figure 5E). These findings are consistent with the in vivo observations and support the coordinated activation of androgen signaling and microglial phagocytic function in females.

Collectively, these results suggest that circulating factors from adolescent HFD-fed females are sufficient to promote microglial activation-like responses, and may contribute to the microglial abnormalities observed in vivo.

### 3.6. Attenuation of Serum-Induced Microglial Activation by AR Blockade in Female Primary Hippocampal Microglia

**Figure 6:**
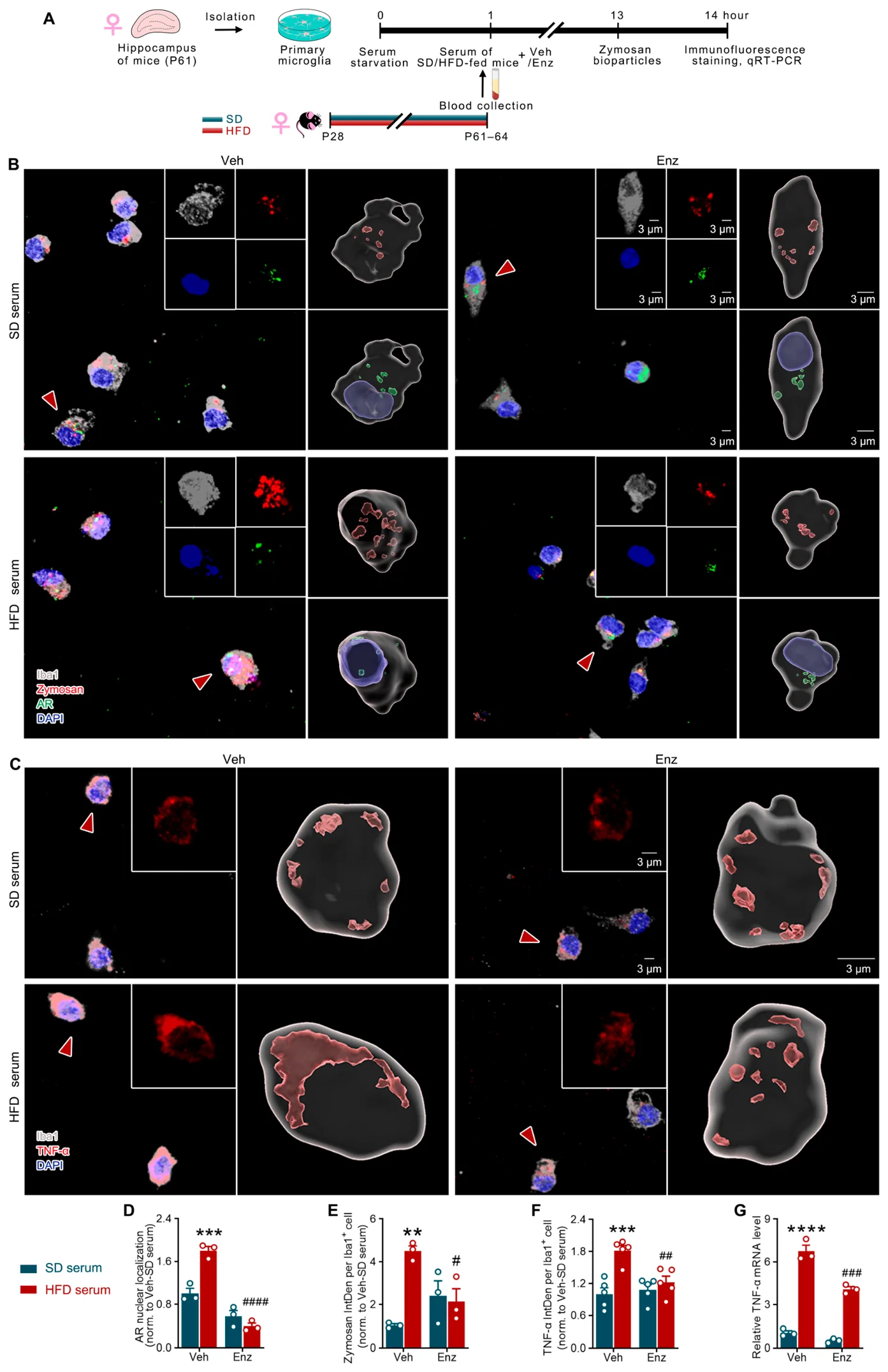
Attenuation of serum-induced microglial activation by AR blockade in female primary hippocampal microglia. (**A**) Design of the in vitro experiment in which female primary hippocampal microglia were treated with serum from female SD- or HFD-fed mice in combination with vehicle control (Veh) or enzalutamide (Enz, an AR antagonist). (**B**) Representative images of immunofluorescence staining for Iba1 (gray), zymosan (red), AR (green), and DAPI (blue) in female primary microglia. High-magnification images showing different fluorescence channels and three-dimensional reconstructions of microglia (gray), zymosan (red), AR (green), and DAPI (blue), as indicated by the arrowheads, are presented. (**C**) Representative images of immunofluorescence staining for Iba1 (gray), TNF-α (red), and DAPI (blue) in female primary microglia. High-magnification images showing different fluorescence channels and three-dimensional reconstructions of microglia (gray) and TNF-α (red), as indicated by the arrowheads, are presented. (**D**,**F**) AR nuclear localization (**D**), the IntDen of zymosan per Iba1^+^ cell (**E**), and the IntDen of TNF-α per Iba1^+^ cell (**F**) in female primary microglia. (**G**) Relative *TNF-α* mRNA levels in female primary microglia. The data are shown as the means ± SEMs. In (**D**–**F**), the data were normalized by dividing each data point by the average of the Veh-SD serum group. *n* = 3–5 independent experiments; two-way ANOVA with Tukey’s post hoc test was performed. ** *p <* 0.01, *** *p <* 0.001, and **** *p <* 0.0001 vs. the Veh-SD serum group; # *p <* 0.05, ## *p <* 0.01, ### *p <* 0.001, and #### *p <* 0.0001 vs. the Veh-HFD serum group.

To further evaluate the contribution of AR signaling to the microglial alterations induced by circulating factors from HFD-fed females, we performed pharmacological inhibition experiments using the AR antagonist enzalutamide (Enz) in primary hippocampal microglia. Female-derived microglia were treated with serum from female HFD- or SD-fed mice in the presence or absence of Enz (Figure 6A).

Consistent with previous findings, serum from HFD-fed females significantly increased AR nuclear localization (Figure 6B,D, *p* = 0.0008) and phagocytic activity (Figure 6B,E, *p* = 0.0038) in microglia compared with serum from SD controls. Notably, Enz treatment effectively abolished the HFD serum-induced increase in AR nuclear localization (Figure 6D, *p* < 0.0001) and significantly reduced microglial phagocytic activity (Figure 6E, *p* = 0.0335), indicating that AR signaling is involved in mediating HFD serum-induced microglial alterations.

We next examined the expression of TNF-α, a downstream signaling molecule associated with AR activation and microglial activation states [71,72]. Both TNF-α protein levels (Figure 6C,F, *p* = 0.0003), as assessed by immunofluorescence, and mRNA levels (Figure 6G, *p* < 0.0001), as measured by qRT–PCR, were significantly elevated in microglia treated with HFD serum. Importantly, these increases were markedly attenuated by Enz at both the protein (Figure 6F, *p* = 0.0057) and transcriptional (Figure 6G, *p* = 0.0003) levels, further supporting the involvement of AR signaling in the regulation of microglial inflammatory responses.

Collectively, these findings provide functional evidence that AR signaling contributes to the microglial activation-like phenotype induced by circulating factors from HFD-fed females. These results, together with the in vivo and serum transfer data, identify microglial androgen signaling as a candidate mechanism linking adolescent metabolic disturbance to alterations in the hippocampus and behavior of females.

## 4. Discussion

The results of the present study demonstrate that adolescent HFD consumption induces anhedonia-like behavior in both sexes, with females displaying broader behavioral impairments and greater behavioral responses to CUMS. These findings are consistent with the marked increase in depression prevalence observed in females after adolescence and support the concept that metabolic disturbances during sensitive developmental periods may influence neurobehavioral vulnerability [3,73].

A major finding of this study is that the behavioral phenotype was accompanied by region-specific hippocampal remodeling, with the most prominent alterations in the vHP and broader reductions in dendritic complexity and spine density in females. Given the central role of the vHP in affective regulation and stress responsiveness [18], these findings suggest that adolescent metabolic disturbances preferentially affect hippocampal circuits involved in emotional processing. Importantly, the stronger interaction between a HFD and CUMS in females further supports the idea that adolescent HFD consumption not only induces behavioral alterations but also increases vulnerability to subsequent environmental stressors.

Our data further identify microglia as a potential cellular interface that links alterations in circulating factors to hippocampal remodeling. Microglia are being increasingly recognized as dynamic regulators of synaptic organization and stress adaptation [74,75], and their activity is highly responsive to peripheral metabolic and endocrine signals [27]. Here, adolescent HFD consumption induced microglial activation-like changes in the vHP of both sexes, with broader changes in soma size and CD68 expression and increased synaptic engulfment detected in females. Notably, these alterations paralleled the observed reductions in dendritic spine density, supporting a possible relationship between excessive microglial phagocytic activity and structural deficits in the hippocampus. Furthermore, chronic stress amplified both microglial activation-like changes and synaptic inclusions in females, suggesting that adolescent HFD consumption may sensitize microglia to subsequent stress-related challenges.

In terms of metabolic parameters, HFD consumption significantly increased body weight and caloric intake in both sexes, despite no significant change in food intake by weight (Figure S10). Although the HFD affected several circulating humoral parameters (FFAs, leptin, and estradiol) in both sexes, circulating testosterone levels increased only in females. Previous studies examining HFD-associated changes in androgen levels have yielded inconsistent findings, likely reflecting differences in developmental stage, dietary paradigm, and sex [35,76]. Nevertheless, the female-specific increase in testosterone detected here suggests that altered androgen signaling may be particularly relevant in females exposed to an adolescent HFD. This possibility is consistent with evidence that females may be more sensitive to deviations in androgen status [77] and with clinical observations linking hyperandrogenemia to an increased risk of depressive symptoms in individuals with conditions such as polycystic ovary syndrome [78].

Mechanistically, our findings support a coordinated relationship between endocrine alterations and microglial inflammatory signaling. HFD-fed females exhibited increases in AR nuclear localization and TNF-α expression in hippocampal microglia, indicating the activation of androgen-responsive proinflammatory pathways. These results are consistent with those of previous studies demonstrating that androgen–AR signaling can modulate immune cell function and promote inflammatory responses [67,71,79]. The findings of the present study suggest that androgen-responsive microglia may link adolescent metabolic challenge to alterations in the hippocampus in females.

Importantly, our serum transfer experiments provide preliminary functional evidence consistent with this model. Circulating factors from HFD-fed females were sufficient to increase AR activation, phagocytic activity, and TNF-α expression in primary female microglia, whereas these effects were attenuated by pharmacological blockade of AR. These findings suggest the involvement of an endocrine-to-microglial signaling axis through which adolescent metabolic disturbances may influence immune functions in the hippocampus. However, because AR manipulation was restricted to in vitro experiments, the contribution of microglial AR signaling to HFD-induced behavioral and structural outcomes in vivo remains unresolved. Thus, while the present findings support a contributory role for androgen-responsive microglial signaling, they do not establish direct causality at the organismal level.

We chose to treat microglia with serum rather than pure testosterone because the aim of the in vitro experiment was to examine the combined influence of the circulating milieu generated by the HFD rather than the effect of a single predefined factor. Serum contains multiple bioactive components, including testosterone, estradiol, FFAs, and leptin. The attenuation of HFD serum-induced microglial responses by enzalutamide supports an AR-dependent component of these effects but does not establish testosterone as the sole active mediator, and contributions from other circulating factors cannot be excluded. Importantly, direct exposure of cultured microglia to serum bypasses the blood–brain barrier and therefore does not recapitulate the in vivo exposure conditions of brain-resident microglia. Nevertheless, this serum transfer approach preserves the complexity of the HFD-associated circulating milieu and provides functional evidence that circulating factors can directly modulate microglial responses in vitro, with AR signaling contributing to this effect.

Interestingly, male mice displayed relative resistance to many of the HFD-induced alterations in microglia and behavior despite physiologically high testosterone levels. This discrepancy suggests that dynamic endocrine responsiveness, rather than absolute hormone concentration alone, may be a critical determinant of neuroimmune sensitivity. In males, testosterone levels are physiologically high and relatively stable [52,77], whereas in females, HFD-induced increases in androgen levels may represent a greater deviation from the baseline endocrine state, thereby producing stronger downstream effects on microglial function and stress responsiveness.

Several limitations of this study should be acknowledged. First, the causal contributions of androgen-responsive microglial signaling to HFD-induced behavioral and hippocampal alterations were not directly validated in vivo. Future studies employing microglia-specific AR manipulation or in vivo pharmacological intervention are needed to determine whether this pathway contributes causally to the observed phenotype. Second, although testosterone has emerged as a candidate mediator, HFD consumption alters the levels of multiple circulating metabolic and endocrine factors, and the relative contribution and potential interactions of these signals remain to be clarified. Third, the present study focused on adolescence as a vulnerable developmental period, but the long-term persistence and reversibility of the observed neuroimmune and neurostructural alterations remain to be determined. Fourth, sample sizes were limited in several experiments, particularly the Golgi morphometric analyses (*n* = 3 mice per group). In addition, although the cellular data were reanalyzed using independent experiments rather than individual cells as the experimental unit to account for the hierarchical structure of the data, the number of independent experiments remained modest (*n* = 3–5). These findings therefore warrant cautious interpretation and replication in larger cohorts. Fifth, analyses were conducted separately by sex, and sex was not included as a factor in the statistical models. Thus, the study characterizes treatment effects within each sex but does not directly test sex differences in these effects. Sixth, the estrous cycle stage was not determined during behavioral testing. Because the present study was designed to examine the effects of HFD consumption within a defined adolescent developmental window, behavioral assessments were performed within the same age range (P56–P60) across sexes and experimental groups after matched durations of dietary and stress exposure [80]. Given the short and individually variable estrous cycle in mice [81], restricting behavioral testing to a single cycle stage would have required a shift in test days for individual females, thereby introducing differences in age and intervention duration. We therefore prioritized temporal matching across experimental groups rather than scheduling behavioral tests according to estrous stage. Nevertheless, cycle-dependent modulation of individual behavioral measures cannot be excluded and should be considered a limitation. Future studies incorporating prospective estrous cycle staging could determine whether specific behavioral responses to adolescent HFD consumption vary across cycle stages. Seventh, the behavioral paradigms used here assess specific depression-related behavioral dimensions in mice, including anhedonia-like behavior and stress coping-related responses, rather than the clinical syndrome of depression. Accordingly, caution is warranted when extrapolating these findings to human depression.

## 5. Conclusions

In conclusion, the results of the present study reveal sex-stratified behavioral and hippocampal neurostructural consequences of adolescent HFD consumption, with females showing broader alterations and a greater behavioral response to stress. HFD consumption increased caloric intake and body weight in both sexes, whereas elevated levels of circulating testosterone and enhanced androgen-responsive microglial signaling were detected in females but not males in the sex-stratified analyses. These findings suggest that androgen-responsive microglial signaling may represent a previously underrecognized candidate link between HFD-associated metabolic disturbance and the broader hippocampal alterations observed in females. However, its causal contribution to the in vivo phenotype remains to be established. Overall, this study provides a framework for further investigating sex-related differences in the neurobiological consequences of adolescent HFD consumption and the potential contribution of endocrine–microglial signaling to these effects.

## Figures and Tables

**Table 1:** Timeline of exposure to the stressors used in the CUMS protocol. The stressors included the following: ① wet bedding for 24 h; ② bedding deprivation for 24 h; ③ 45° cage tilting for 14 h; ④ cage shaking for 30 min; ⑤ physical restraint for 2 h; ⑥ forced swimming at 8 °C for 1 min; and ⑦ tail nipping (1 cm from the tip of the tail) for 1 min.

Day	Stressor	Day	Stressor	Day	Stressor	Day	Stressor
P42	①⑤	P47	④⑥	P52	①④	P57	②⑥
P43	②④	P48	③⑦	P53	②⑤	P58	③⑦
P44	③⑥	P49	①⑤	P54	③⑥	P59	①⑤
P45	①⑦	P50	②⑥	P55	①⑦	P60	④⑥
P46	②⑤	P51	③⑦	P56	④⑤		

**Table 2:** qRT–PCR primers used in this study.

Gene	Forward Primer (5′–3′)	Reverse Primer (5′–3′)
*TNF-α*	CAGGCGGTGCCTATGTCTC	CGATCACCCCGAAGTTCAGTAG
*β-actin*	GGCTGTATTCCCCTCCATCG	CCAGTTGGTAACAATGCCATGT

## Data Availability

The original contributions presented in this study are included in the article/Supplementary Materials. Further inquiries can be directed to the corresponding author.

## Supplementary Materials

The following supporting information can be downloaded at: https://www.mdpi.com/article/10.3390/nu18193229/s1, Figure S1: Representative images of vaginal cytology for each estrous cycle stage; Figure S2: The purity of primary microglia; Figure S3: The viability of primary microglia; Figure S4: Localization of hippocampal ROIs; Figure S5: Sex-stratified characterization of locomotor distance and total liquid intake responses to adolescent HFD consumption in mice; Figure S6: Sex-stratified characterization of dorsal hippocampal neurostructural responses to adolescent HFD consumption in mice; Figure S7: Sex-stratified characterization of dorsal hippocampal microglial responses to adolescent HFD consumption in mice; Figure S8: Sex-stratified analyses of serum, microglial, and behavioral parameters; Figure S9: Sex-stratified analyses of serum testosterone levels, microglial androgen signaling, microglial parameters, and depression-like behaviors; Figure S10: Sex-stratified characterization of body weight, food consumption and energy intake responses to adolescent HFD consumption in mice; Table S1: List of the statistical results in Figure 1; Table S2: List of the statistical results in Figure 2; Table S3: List of the statistical results in Figure 3; Table S4: List of the statistical results in Figure 6; Table S5: List of the statistical results in Figure S5; Table S6: List of the statistical results in Figure S6; Table S7: List of the statistical results in Figure S7; Table S8: List of the statistical results in Figure S10.
