# Supplementary material for: Sex-Stratified Characterization of Behavioral, Hippocampal, and Microglial Androgen Responses to Adolescent High-Fat Diet Consumption in Mice
Source: Nutrients. 2026 Sep 30;18(19):3229. doi: 10.3390/nu18193229 (PMC13666788; doi:10.3390/nu18193229)
Supplement: Supplementary file 1 [file nutrients-18-03229-s001.zip › nutrients-4485893-supplementary.pdf]

Supplementary Information

Sex-Stratified Characterization of Behavioral, Hippocampal, and Microglial Androgen Responses to Adolescent High-Fat Diet Consumption in Mice

Conghui Wang [1], Hong Li [2], Zhehao Yu [1], Bingjie Xie [1], Xiuting Yao [1], Jingyi Zhao [2], Yuxi Chen [1], Hong Zhuang [1], Chongyang Yang [1] and Lijie Liu [3,*]

[1] Medical College, Southeast University, Nanjing 210009, China; jnconghui2009@126.com (C.W.); 17551120513@163.com (Z.Y.); 220234159@seu.edu.cn (B.X.); delia415171022@163.com (X.Y.); 230239144@seu.edu.cn (Y.C.); 101300426@seu.edu.cn (H.Z.); 220244350@seu.edu.cn (C.Y.)

[2] School of Life Science and Technology, Southeast University, Nanjing 210009, China; 13203929028@163.com (H.L.); zjysk175@163.com (J.Z.)

[3] Jiangsu Provincial Key Laboratory of Critical Care Medicine, Department of Physiology, School of Medicine, Southeast University, Nanjing 210009, China

* Correspondence: liulijie@seu.edu.cn

Supplementary Figures

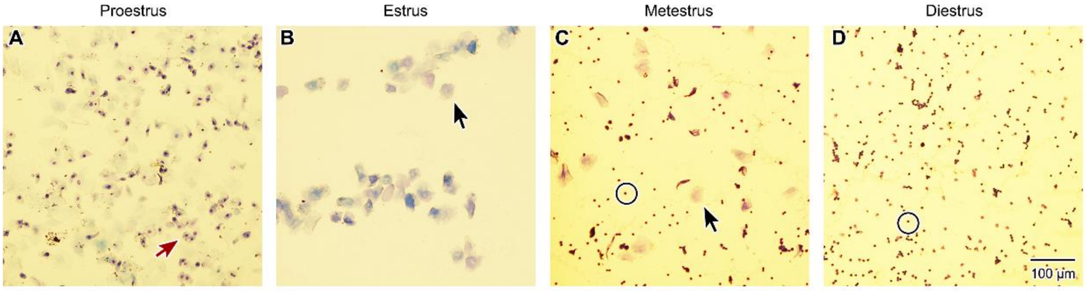

Figure S1. Representative images of vaginal cytology for each estrous cycle stage. **A-D** Stages of estrous cycle include proestrus(**A**), estrus (**B**), metestrus (**C**), and diestrus (**D**). The red arrow, black arrow, and circle indicate nucleated epithelial cells, cornified epithelial cells, and leukocytes, respectively.

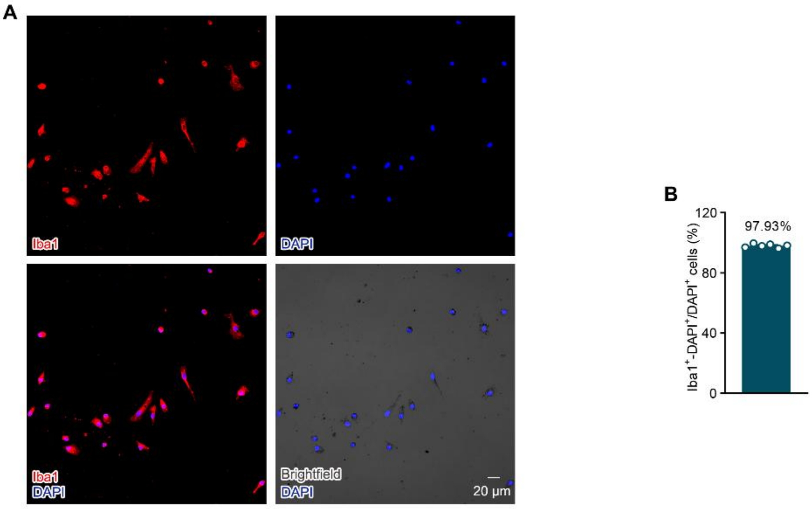

Figure S2. The purity of primary microglia. **A** Representative images of Iba1 (red) and DAPI (blue) immunofluorescence staining along with corresponding brightfield images. **B** Proportion of Iba1^{+}-DAPI^{+} cells among total DAPI^{+} cells. The data are shown as the means ± SEMs. n = 6 independent experiments.

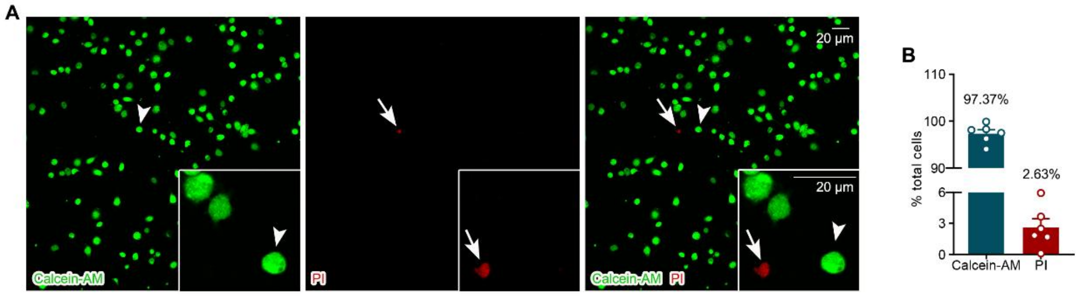

Figure S3. The viability of primary microglia. **A** Representative images of primary microglia stained with calcein-AM (green, live cells) and PI (red, dead cells). Insets show higher magnification images of live (green, arrowheads) and dead (red, arrows) cells. **B** Proportion of calcein-AM$^+$ cells and PI$^+$ cells among total cells. The data are shown as the means ± SEMs. n = 6 independent experiments.

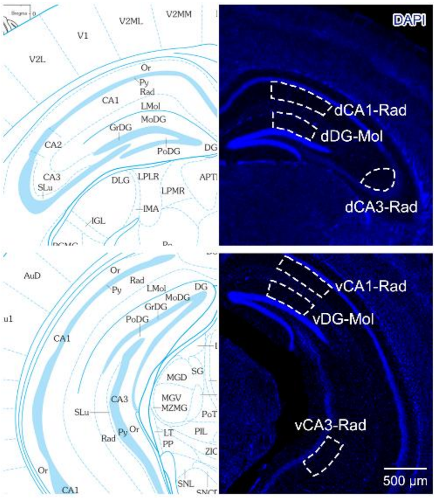

Figure S4. Localization of hippocampal ROIs. Left: Dorsal (top panel, bregma −2.30 mm) and ventral (bottom panel, bregma −3.08 mm) hippocampal ROIs in mouse brain atlas [1]. Right panel: Images of coronal brain sections with DAPI staining, with the corresponding mask of the mouse brain atlas presented on the left. The dashed outlines indicate the ROIs used for analysis, including the DG molecular layer (dDG-Mol, vDG-Mol), CA3 stratum radiatum (dCA3-Rad, vCA3-Rad), and CA1 stratum radiatum

(dCA1-Rad, vCA1-Rad) of both the dorsal (top panel) and ventral (bottom panel) hippocampus.

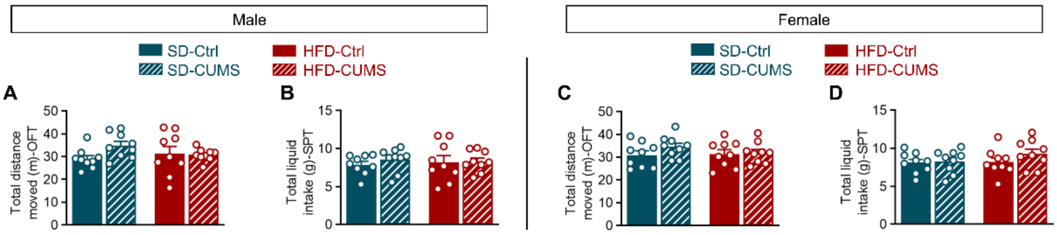

Figure S5. Sex-stratified characterization of locomotor distance and total liquid intake responses to adolescent HFD consumption in mice. **A** and **C** Total distance traveled in the OFT for male and female mice. **B** and **D** Total liquid intake during the SPT for male and female mice. n =9 mice per group. The data were analyzed separately for each sex by two-way ANOVA with diet and stress as factors, followed by Tukey's post hoc test where appropriate. Significant main effects of diet or stress and diet × stress interactions are indicated above the corresponding panels as "diet", "stress", or "interaction", respectively; only statistically significant effects are shown.

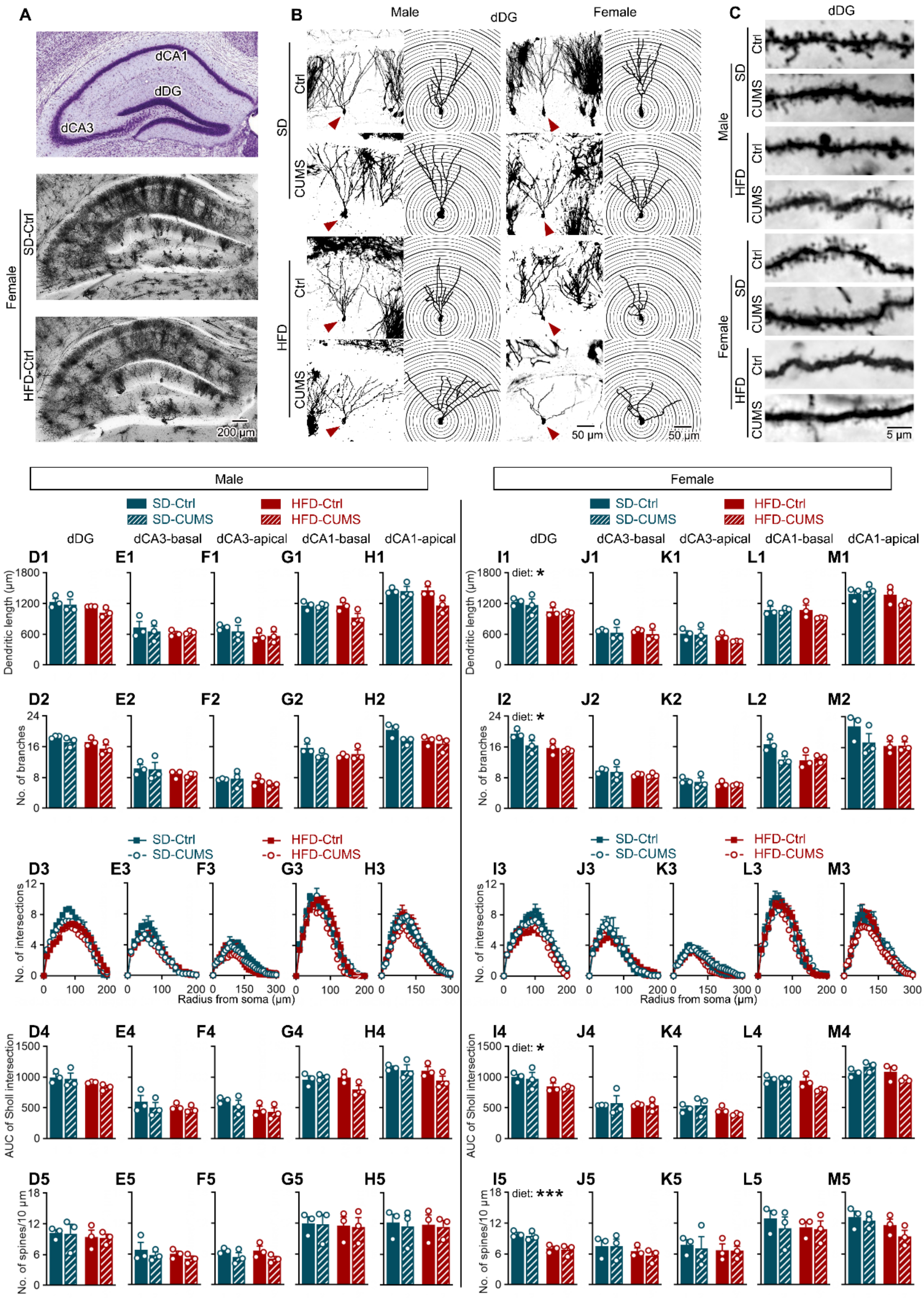

Figure S6. Sex-stratified characterization of dorsal hippocampal neurostructural responses to adolescent HFD consumption in mice. A Top panel: ROIs in the dorsal

hippocampus in the mouse brain atlas [1]. Representative images of Golgi-stained cells in the dorsal hippocampi of female SD-Ctrl (middle panel) and HFD-Ctrl (bottom panel) mice. **B** Representative Golgi-stained granule cells in the dDG region (indicated by red arrowheads, left) and their traces along with a graphic description of the Sholl analysis parameters (right). **C** Representative photomicrographs of the secondary branches of granule cells in the dDG region. **D–M** Dendritic lengths (**D1–M1**), numbers of dendritic branches (**D2–M2**), numbers of dendritic intersections with the equidistant Sholl spheres (**D3–M3**), AUCs of the Sholl intersection (**D4–M4**), and numbers of dendritic spines/10 μm (**D5–M5**) in the ROIs of male and female mice. The data are shown as the means ± SEMs. n = 3 mice per group. The data were analyzed separately for each sex by two-way ANOVA with diet and stress as factors, followed by Tukey's post hoc test where appropriate. Significant main effects of diet or stress and diet × stress interactions are indicated above the corresponding panels as "diet", "stress", or "interaction", respectively; only statistically significant effects are shown. * $P < 0.05$, *** $P < 0.001$ indicate the significance level of the corresponding main effect or interaction.

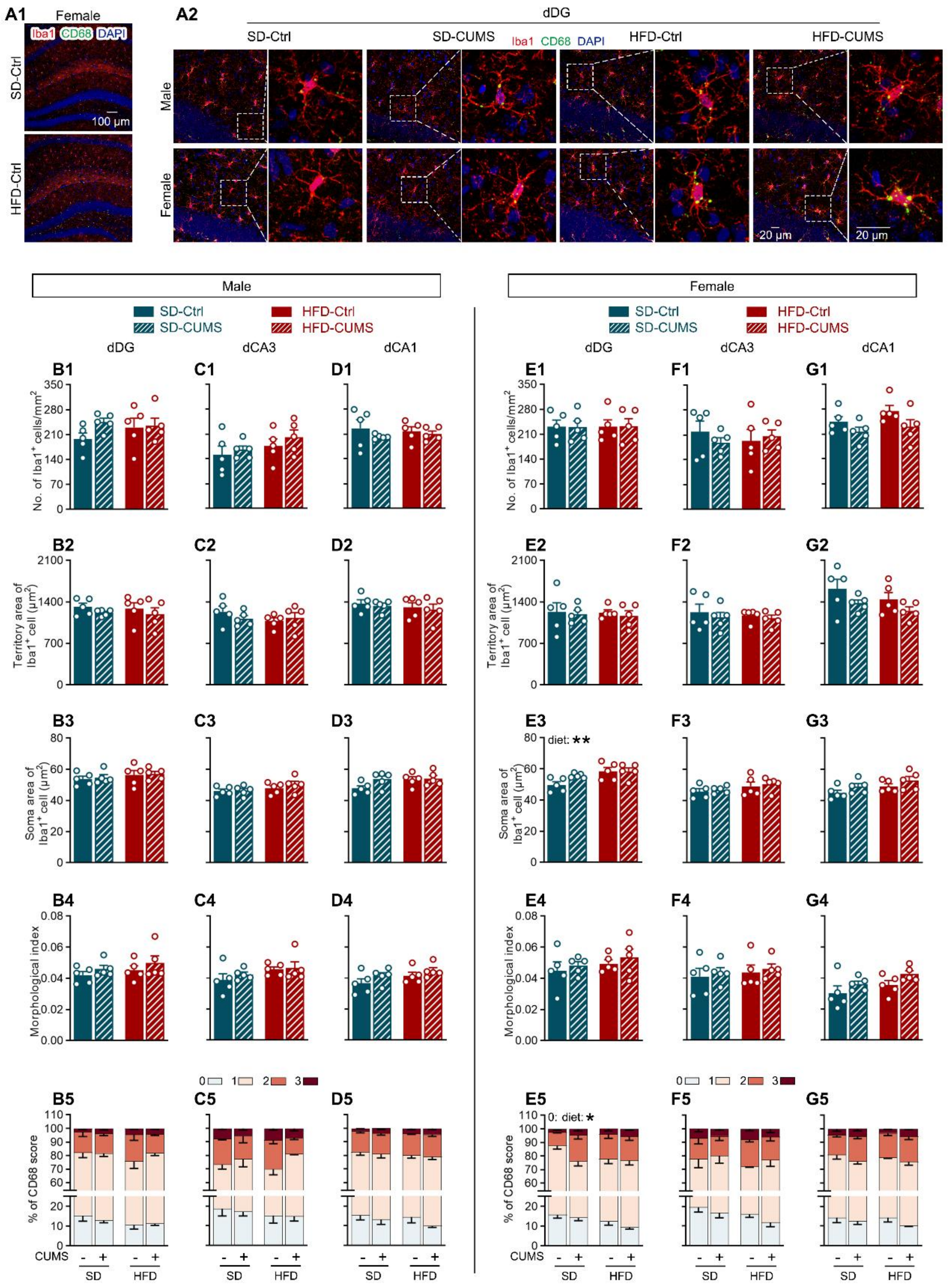

Figure S7. Sex-stratified characterization of dorsal hippocampal microglial responses to adolescent HFD consumption in mice. A1 Representative low-magnification images of immunofluorescence staining for Iba1 (red), CD68 (green),

and DAPI (blue) in the dorsal hippocampi of female SD-Ctrl and HFD-Ctrl mice (10× magnification). **A2** Representative high-magnification images of immunofluorescence staining for Iba1 (red), CD68 (green), and DAPI (blue) in the dDG regions of all groups (40× magnification). Areas indicated by dashed lines are shown at higher magnification on the right. **B–G** Microglial density (**B1–G1**), territory area (**B2–G2**), soma area (**B3–G3**), morphological index (**B4–G4**), and percentage of microglia with $CD68^{+}$ scores ranging from 0 to 3 (**B5–G5**) in the ROIs of male and female mice. – and + indicate the Ctrl and CUMS subgroups, respectively. The data are shown as the means ± SEMs. n = 5 mice per group. The data were analyzed separately for each sex by two-way ANOVA with diet and stress as factors, followed by Tukey's post hoc test where appropriate. Significant main effects of diet or stress and diet × stress interactions are indicated above the corresponding panels as "diet", "stress", or "interaction", respectively; only statistically significant effects are shown. * $P < 0.05$, ** $P < 0.01$ indicate the significance level of the corresponding main effect or interaction.

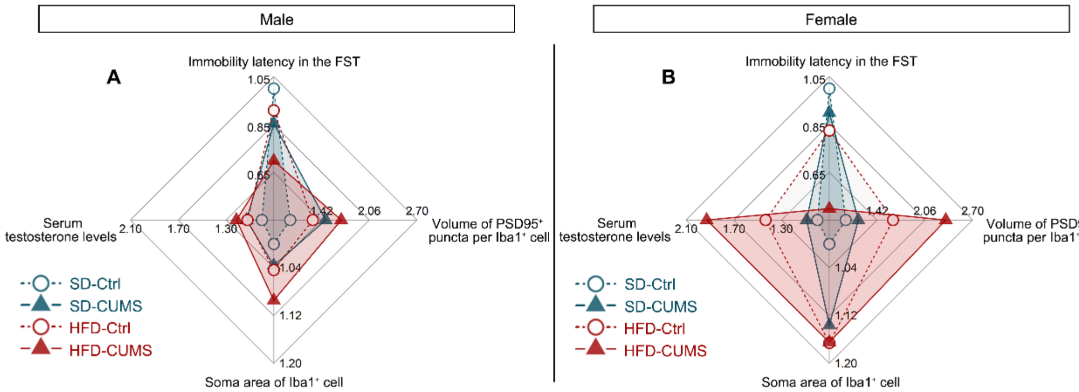

Figure S8. Sex-stratified analyses of serum, microglial, and behavioral parameters.

A and **B** Radar charts depicting the serum, microglial (vDG region), and behavioral

parameters of male and female mice. The data were normalized by dividing each data point by the average of the sex-matched SD-Ctrl group. The data are shown as the means. n = 5 mice per group.

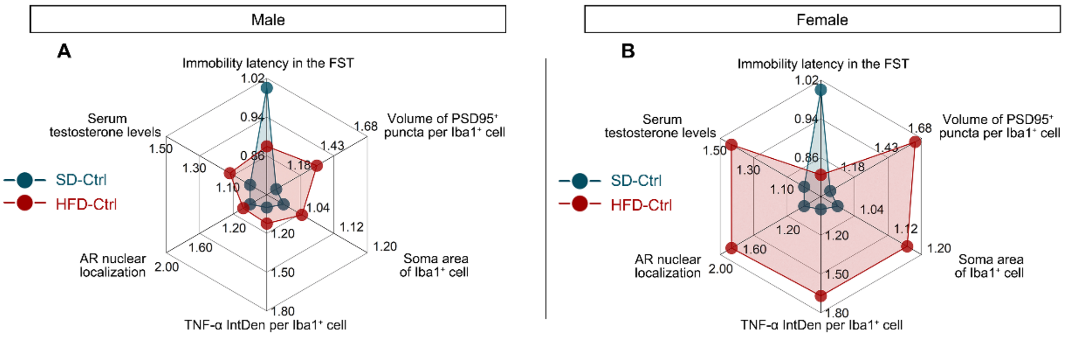

Figure S9. Sex-stratified analyses of serum testosterone levels, microglial androgen signaling, microglial parameters, and depression-like behaviors. A and **B** Radar charts depicting the serum, microglial (vDG region), and behavioral parameters of male and female mice. The data were normalized by dividing each data point by the average of the sex-matched SD-Ctrl group. The data are shown as the means. n = 5 mice per group.

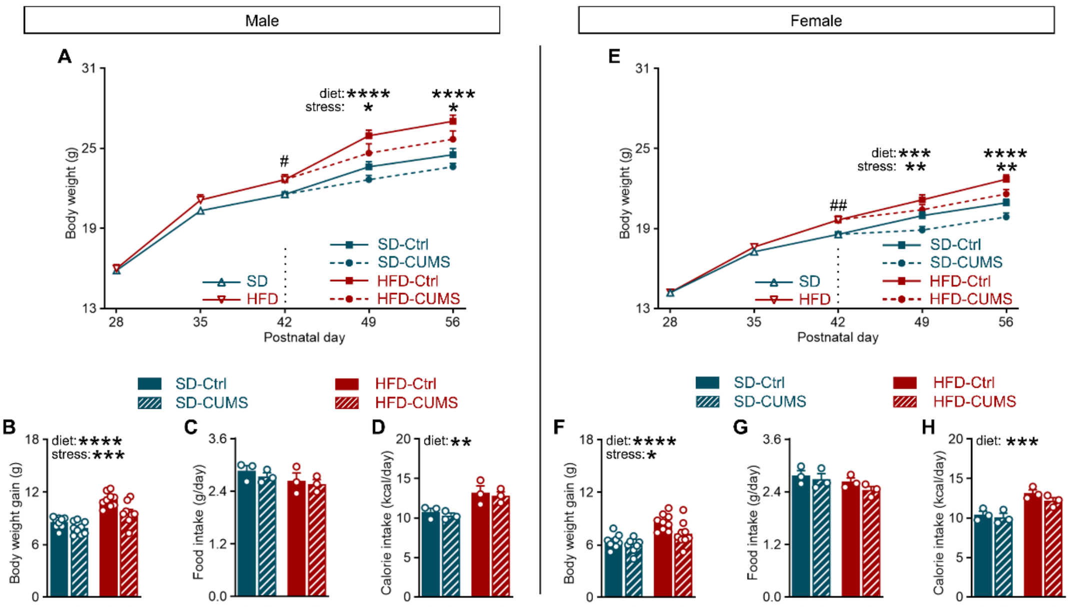

Figure S10. Sex-stratified characterization of body weight, food consumption and energy intake responses to adolescent HFD consumption in mice. **A-H** Body weight (**A, E**), body weight gain **(B, F**), food intake by weight (**C, G**) and caloric intake (**D, H**) of male and female mice. The data are shown as the means ± SEMs. In (A, B, E, and F), n = 18 mice per group for the SD and HFD groups, and n = 9 mice per subgroup for the SD-Ctrl, HFD-Ctrl, SD-CUMD, and HFD-CUMD groups. In (C, D, G, and H), n = 3 cages per group, with 3 mice per cage. The data were analyzed separately for each sex by Student's t test or two-way ANOVA with diet and stress as factors, followed by Tukey's post hoc test where appropriate. Significant main effects of diet or stress and diet × stress interactions are indicated above the corresponding panels as "diet", "stress", or "interaction", respectively; only statistically significant effects and comparisons are shown. # $P < 0.05$, ## $P < 0.01$ vs. the sex-matched SD group (Student's t test). * $P < 0.05$, ** $P < 0.01$, *** $P < 0.001$, and **** $P < 0.0001$ indicate the significance level of the corresponding main effect or interaction.

Supplementary Tables

Figure	Statistical values	Main effect	*P* value	Post hoc tests
1C	$Diet_{(SD;HFD)}$ $F_{(1,32)} = 2.050$	No	0.1619	
	$Stress_{(Ctrl;CUMS)}$ $F_{(1,32)} = 0.1223$	No	0.7288	
	Interaction $F_{(1,32)} = 1.102$	No	0.3016	
1E	$Diet_{(SD;HFD)}$ $F_{(1,32)} = 0.1043$	No	0.7488	
	$Stress_{(Ctrl;CUMS)}$ $F_{(1,32)} = 53.77$	Yes	<0.0001	
	Interaction $F_{(1,32)} = 0.0234$	No	0.8794	
1F	$Diet_{(SD;HFD)}$ $F_{(1,32)} = 3.750$	No	0.0617	
	$Stress_{(Ctrl;CUMS)}$ $F_{(1,32)} = 8.872$	Yes	0.0055	
	Interaction $F_{(1,32)} = 0.2714$	No	0.6059	
1G	$Diet_{(SD;HFD)}$ $F_{(1,32)} = 4.237$	Yes	0.0478	
	$Stress_{(Ctrl;CUMS)}$ $F_{(1,32)} = 0.7948$	No	0.3793	
	Interaction $F_{(1,32)} = 0.4788$	No	0.4940	

(Table continues.)

Figure	Statistical values	Main effect	*P* value	Post hoc tests
1H	Diet$_{(SD;HFD)}$ $F_{(1,28)} = 3.301$	No	0.0799	SD-Ctrl vs. HFD-Ctrl: $P = 0.0011$
	Stress$_{(Ctrl;CUMS)}$ $F_{(1,28)} = 9.307$	Yes	0.0050	
	Interaction $F_{(1,28)} = 17.72$	Yes	0.0002	
1I	Diet$_{(SD;HFD)}$ $F_{(1,25)} = 17.99$	Yes	0.0003	
	Stress$_{(Ctrl;CUMS)}$ $F_{(1,25)} = 32.24$	Yes	<0.0001	
	Interaction $F_{(1,25)} = 12.53$	Yes	0.0016	
1J	Diet$_{(SD;HFD)}$ $F_{(1,32)} = 6.570$	Yes	0.0153	
	Stress$_{(Ctrl;CUMS)}$ $F_{(1,32)} = 0.1571$	No	0.6945	
	Interaction $F_{(1,32)} = 7.079$	Yes	0.0121	
1K	Diet$_{(SD;HFD)}$ $F_{(1,32)} = 0.0016$	No	0.9680	
	Stress$_{(Ctrl;CUMS)}$ $F_{(1,32)} = 0.2366$	No	0.6300	
	Interaction $F_{(1,32)} = 0.6616$	No	0.4220	

(Table continues.)

Figure	Statistical values	Main effect	P value	Post hoc tests
1M	Diet$_{(SD;HFD)}$	Yes	0.0004	
	$F_{(1,32)} = 15.86$			
	Stress$_{(Ctrl;CUMS)}$	No	0.3420	
	$F_{(1,32)} = 0.9303$			
	Interaction	No	0.2282	
	$F_{(1,32)} = 1.509$			
1O	Diet$_{(SD;HFD)}$	Yes	0.0001	
	$F_{(1,32)} = 18.89$			
	Stress$_{(Ctrl;CUMS)}$	Yes	<0.0001	
	$F_{(1,32)} = 113.6$			
	Interaction	No	0.9421	
	$F_{(1,32)} = 0.0054$			
1P	Diet$_{(SD;HFD)}$	Yes	<0.0001	SD-CUMS vs. HFD-CUMS: $P < 0.0001$
	$F_{(1,32)} = 28.23$			
	Stress$_{(Ctrl;CUMS)}$	Yes	0.0015	
	$F_{(1,32)} = 12.00$			
	Interaction	Yes	0.0053	
	$F_{(1,32)} = 8.966$			
1Q	Diet$_{(SD;HFD)}$	Yes	<0.0001	
	$F_{(1,32)} = 33.10$			
	Stress$_{(Ctrl;CUMS)}$	No	0.1553	
	$F_{(1,32)} = 2.118$			
	Interaction	No	0.1814	
	$F_{(1,32)} = 1.867$			

(Table continues.)

Figure	Statistical values	Main effect	*P* value	Post hoc tests
1R	Diet$_{(SD;HFD)}$ $F_{(1,28)} = 2.630$	No	0.1161	SD-Ctrl vs. HFD-Ctrl: *P* = 0.0090
	Stress$_{(Ctrl;CUMS)}$ $F_{(1,28)} = 7.186$	Yes	0.0122	
	Interaction $F_{(1,28)} = 10.66$	Yes	0.0029	
1S	Diet$_{(SD;HFD)}$ $F_{(1,22)} = 11.95$	Yes	0.0022	
	Stress$_{(Ctrl;CUMS)}$ $F_{(1,22)} = 7.160$	Yes	0.0138	
	Interaction $F_{(1,22)} = 5.938$	Yes	0.0234	
1T	Diet$_{(SD;HFD)}$ $F_{(1,30)} = 9.840$	Yes	0.0038	
	Stress$_{(Ctrl;CUMS)}$ $F_{(1,30)} = 0.3555$	No	0.5555	
	Interaction $F_{(1,30)} = 12.26$	Yes	0.0015	
1U	Diet$_{(SD;HFD)}$ $F_{(1,32)} = 49.46$	Yes	<0.0001	
	Stress$_{(Ctrl;CUMS)}$ $F_{(1,32)} = 2.594$	No	0.1171	
	Interaction $F_{(1,32)} = 0.3092$	No	0.5821	

Table S1: List of the statistical results in Figure 1.

Figure	Statistical values	Main effect	*P* value	Post hoc tests
2D1	Diet$_{(SD;HFD)}$	No	0.2596	
	$F_{(1,8)} = 1.473$			
	Stress$_{(Ctrl;CUMS)}$	No	0.3998	
	$F_{(1,8)} = 0.791$			
	Interaction	No	0.3418	
	$F_{(1,8)} = 1.021$			
2D2	Diet$_{(SD;HFD)}$	No	0.4917	
	$F_{(1,8)} = 0.5193$			
	Stress$_{(Ctrl;CUMS)}$	No	0.0835	
	$F_{(1,8)} = 3.905$			
	Interaction	No	0.3413	
	$F_{(1,8)} = 1.023$			
2D4	Diet$_{(SD;HFD)}$	No	0.0545	
	$F_{(1,8)} = 5.064$			
	Stress$_{(Ctrl;CUMS)}$	No	0.5150	
	$F_{(1,8)} = 0.4639$			
	Interaction	No	0.3790	
	$F_{(1,8)} = 0.8671$			
2D5	Diet$_{(SD;HFD)}$	No	0.1396	
	$F_{(1,8)} = 2.690$			
	Stress$_{(Ctrl;CUMS)}$	Yes	0.0025	
	$F_{(1,8)} = 18.84$			
	Interaction	No	0.1841	
	$F_{(1,8)} = 2.113$			

(Table continues.)

Figure	Statistical values	Main effect	*P* value	Post hoc tests
2E1	Diet$_{(SD;HFD)}$	No	0.1606	
	$F_{(1,8)} = 2.391$			
	Stress$_{(Ctrl;CUMS)}$	No	0.1048	
	$F_{(1,8)} = 3.345$			
	Interaction	No	0.3653	
	$F_{(1,8)} = 0.921$			
2E2	Diet$_{(SD;HFD)}$	No	0.1317	
	$F_{(1,8)} = 2.818$			
	Stress$_{(Ctrl;CUMS)}$	No	0.1897	
	$F_{(1,8)} = 2.054$			
	Interaction	No	0.3215	
	$F_{(1,8)} = 1.117$			
2E4	Diet$_{(SD;HFD)}$	No	0.1162	
	$F_{(1,8)} = 3.102$			
	Stress$_{(Ctrl;CUMS)}$	No	0.0853	
	$F_{(1,8)} = 3.853$			
	Interaction	No	0.2518	
	$F_{(1,8)} = 1.526$			
2E5	Diet$_{(SD;HFD)}$	No	0.3165	
	$F_{(1,8)} = 1.141$			
	Stress$_{(Ctrl;CUMS)}$	No	0.7238	
	$F_{(1,8)} = 0.1341$			
	Interaction	No	0.9280	
	$F_{(1,8)} = 0.0087$			

(Table continues.)

Figure	Statistical values	Main effect	*P* value	Post hoc tests
2F1	Diet$_{(SD;HFD)}$	No	0.2517	
	$F_{(1,8)} = 1.527$			
	Stress$_{(Ctrl;CUMS)}$	No	0.8703	
	$F_{(1,8)} = 0.028$			
	Interaction	No	0.5913	
	$F_{(1,8)} = 0.3128$			
2F2	Diet$_{(SD;HFD)}$	No	0.1555	
	$F_{(1,8)} = 2.459$			
	Stress$_{(Ctrl;CUMS)}$	No	0.2008	
	$F_{(1,8)} = 1.944$			
	Interaction	No	0.1296	
	$F_{(1,8)} = 2.854$			
2F4	Diet$_{(SD;HFD)}$	No	0.2771	
	$F_{(1,8)} = 1.360$			
	Stress$_{(Ctrl;CUMS)}$	No	0.8936	
	$F_{(1,8)} = 0.019$			
	Interaction	No	0.8417	
	$F_{(1,8)} = 0.043$			
2F5	Diet$_{(SD;HFD)}$	No	0.3865	
	$F_{(1,8)} = 0.8390$			
	Stress$_{(Ctrl;CUMS)}$	No	0.6333	
	$F_{(1,8)} = 0.2459$			
	Interaction	No	0.9652	
	$F_{(1,8)} = 0.0020$			

(Table continues.)

Figure	Statistical values	Main effect	*P* value	Post hoc tests
2G1	Diet$_{(SD;HFD)}$	No	0.7440	
	$F_{(1,8)} = 0.1143$			
	Stress$_{(Ctrl;CUMS)}$	No	0.8485	
	$F_{(1,8)} = 0.0390$			
	Interaction	No	0.7408	
	$F_{(1,8)} = 0.1173$			
2G2	Diet$_{(SD;HFD)}$	No	0.8930	
	$F_{(1,8)} = 0.0193$			
	Stress$_{(Ctrl;CUMS)}$	No	0.5927	
	$F_{(1,8)} = 0.3103$			
	Interaction	No	0.6387	
	$F_{(1,8)} = 0.2380$			
2G4	Diet$_{(SD;HFD)}$	No	0.6042	
	$F_{(1,8)} = 0.2910$			
	Stress$_{(Ctrl;CUMS)}$	No	0.6730	
	$F_{(1,8)} = 0.1918$			
	Interaction	No	0.6125	
	$F_{(1,8)} = 0.2776$			
2G5	Diet$_{(SD;HFD)}$	No	0.2123	
	$F_{(1,8)} = 1.838$			
	Stress$_{(Ctrl;CUMS)}$	No	0.6449	
	$F_{(1,8)} = 0.2292$			
	Interaction	No	0.9991	
	$F_{(1,8)} < 0.0001$			

(Table continues.)

Figure	Statistical values	Main effect	*P* value	Post hoc tests
2H1	$Diet_{(SD;HFD)}$	Yes	0.0239	
	$F_{(1,8)} = 7.728$			
	$Stress_{(Ctrl;CUMS)}$	No	0.0854	
	$F_{(1,8)} = 3.850$			
	Interaction	No	0.2025	
	$F_{(1,8)} = 1.927$			
2H2	$Diet_{(SD;HFD)}$	No	0.0643	
	$F_{(1,8)} = 4.600$			
	$Stress_{(Ctrl;CUMS)}$	Yes	0.0255	
	$F_{(1,8)} = 7.495$			
	Interaction	No	0.4612	
	$F_{(1,8)} = 0.599$			
2H4	$Diet_{(SD;HFD)}$	Yes	0.0217	
	$F_{(1,8)} = 8.088$			
	$Stress_{(Ctrl;CUMS)}$	No	0.1168	
	$F_{(1,8)} = 3.091$			
	Interaction	No	0.3305	
	$F_{(1,8)} = 1.073$			
2H5	$Diet_{(SD;HFD)}$	No	0.5697	
	$F_{(1,8)} = 0.3514$			
	$Stress_{(Ctrl;CUMS)}$	No	0.4122	
	$F_{(1,8)} = 0.7484$			
	Interaction	No	0.8040	
	$F_{(1,8)} = 0.0658$			

(Table continues.)

Figure	Statistical values	Main effect	*P* value	Post hoc tests
2I1	Diet$_{(SD;HFD)}$	Yes	0.0002	
	$F_{(1,8)} = 41.60$			
	Stress$_{(Ctrl;CUMS)}$	Yes	0.0334	
	$F_{(1,8)} = 6.581$			
	Interaction	No	0.9586	
	$F_{(1,8)} = 0.003$			
2I2	Diet$_{(SD;HFD)}$	Yes	0.0001	SD-CUMS vs. HFD-CUMS: *P* = 0.0475
	$F_{(1,8)} = 48.60$			
	Stress$_{(Ctrl;CUMS)}$	Yes	0.0005	
	$F_{(1,8)} = 31.86$			
	Interaction	Yes	0.0438	
	$F_{(1,8)} = 5.716$			
2I4	Diet$_{(SD;HFD)}$	Yes	0.0005	
	$F_{(1,8)} = 32.58$			
	Stress$_{(Ctrl;CUMS)}$	No	0.0765	
	$F_{(1,8)} = 4.134$			
	Interaction	No	0.8890	
	$F_{(1,8)} = 0.021$			
2I5	Diet$_{(SD;HFD)}$	Yes	0.0041	
	$F_{(1,8)} = 15.83$			
	Stress$_{(Ctrl;CUMS)}$	Yes	0.0074	
	$F_{(1,8)} = 12.67$			
	Interaction	No	0.3403	
	$F_{(1,8)} = 1.028$			

(Table continues.)

Figure	Statistical values	Main effect	*P* value	Post hoc tests
2J1	Diet$_{(SD;HFD)}$	No	0.5164	
	$F_{(1,8)} = 0.461$			
	Stress$_{(Ctrl;CUMS)}$	No	0.2867	
	$F_{(1,8)} = 1.303$			
	Interaction	No	0.8723	
	$F_{(1,8)} = 0.028$			
2J2	Diet$_{(SD;HFD)}$	No	0.1306	
	$F_{(1,8)} = 2.837$			
	Stress$_{(Ctrl;CUMS)}$	No	0.0665	
	$F_{(1,8)} = 4.509$			
	Interaction	No	0.1296	
	$F_{(1,8)} = 2.855$			
2J4	Diet$_{(SD;HFD)}$	No	0.4407	
	$F_{(1,8)} = 0.6580$			
	Stress$_{(Ctrl;CUMS)}$	No	0.5434	
	$F_{(1,8)} = 0.4027$			
	Interaction	No	0.6177	
	$F_{(1,8)} = 0.2696$			
2J5	Diet$_{(SD;HFD)}$	No	0.3482	
	$F_{(1,8)} = 0.9931$			
	Stress$_{(Ctrl;CUMS)}$	No	0.1965	
	$F_{(1,8)} = 1.985$			
	Interaction	No	0.5285	
	$F_{(1,8)} = 0.4340$			

(Table continues.)

Figure	Statistical values	Main effect	*P* value	Post hoc tests
2K1	$Diet_{(SD;HFD)}$	No	0.4480	
	$F_{(1,8)} = 0.6367$			
	$Stress_{(Ctrl;CUMS)}$	No	0.1974	
	$F_{(1,8)} = 1.976$			
	Interaction	No	0.6266	
	$F_{(1,8)} = 0.2559$			
2K2	$Diet_{(SD;HFD)}$	Yes	0.0214	
	$F_{(1,8)} = 8.129$			
	$Stress_{(Ctrl;CUMS)}$	Yes	0.0300	
	$F_{(1,8)} = 6.933$			
	Interaction	No	0.3322	
	$F_{(1,8)} = 1.065$			
2K4	$Diet_{(SD;HFD)}$	No	0.3816	
	$F_{(1,8)} = 0.8574$			
	$Stress_{(Ctrl;CUMS)}$	No	0.3204	
	$F_{(1,8)} = 1.122$			
	Interaction	No	0.5011	
	$F_{(1,8)} = 0.4962$			
2K5	$Diet_{(SD;HFD)}$	No	0.0700	
	$F_{(1,8)} = 4.371$			
	$Stress_{(Ctrl;CUMS)}$	No	0.1452	
	$F_{(1,8)} = 2.605$			
	Interaction	No	0.3892	
	$F_{(1,8)} = 0.8288$			

(Table continues.)

Figure	Statistical values	Main effect	*P* value	Post hoc tests
2L1	$Diet_{(SD;HFD)}$	Yes	0.0414	
	$F_{(1,8)} = 5.892$			
	$Stress_{(Ctrl;CUMS)}$	No	0.1553	
	$F_{(1,8)} = 2.461$			
	Interaction	No	0.9395	
	$F_{(1,8)} = 0.006$			
2L2	$Diet_{(SD;HFD)}$	No	0.6430	
	$F_{(1,8)} = 0.2319$			
	$Stress_{(Ctrl;CUMS)}$	No	0.2856	
	$F_{(1,8)} = 1.309$			
	Interaction	No	0.9595	
	$F_{(1,8)} = 0.003$			
2L4	$Diet_{(SD;HFD)}$	Yes	0.0317	
	$F_{(1,8)} = 6.751$			
	$Stress_{(Ctrl;CUMS)}$	No	0.4218	
	$F_{(1,8)} = 0.7167$			
	Interaction	No	0.6842	
	$F_{(1,8)} = 0.1780$			
2L5	$Diet_{(SD;HFD)}$	Yes	0.0207	
	$F_{(1,8)} = 8.264$			
	$Stress_{(Ctrl;CUMS)}$	No	0.2532	
	$F_{(1,8)} = 1.516$			
	Interaction	No	0.3415	
	$F_{(1,8)} = 1.023$			

(Table continues.)

Figure	Statistical values	Main effect	*P* value	Post hoc tests
2M1	$Diet_{(SD;HFD)}$	Yes	0.0177	
	$F_{(1,8)} = 8.867$			
	$Stress_{(Ctrl;CUMS)}$	No	0.5123	
	$F_{(1,8)} = 0.4701$			
	Interaction	No	0.6450	
	$F_{(1,8)} = 0.2291$			
2M2	$Diet_{(SD;HFD)}$	No	0.1236	
	$F_{(1,8)} = 2.960$			
	$Stress_{(Ctrl;CUMS)}$	No	0.7874	
	$F_{(1,8)} = 0.0778$			
	Interaction	No	0.3654	
	$F_{(1,8)} = 0.9207$			
2M4	$Diet_{(SD;HFD)}$	Yes	0.0177	
	$F_{(1,8)} = 8.849$			
	$Stress_{(Ctrl;CUMS)}$	No	0.6211	
	$F_{(1,8)} = 0.2643$			
	Interaction	No	0.5995	
	$F_{(1,8)} = 0.2989$			
2M5	$Diet_{(SD;HFD)}$	Yes	0.0002	
	$F_{(1,8)} = 40.14$			
	$Stress_{(Ctrl;CUMS)}$	No	0.0765	
	$F_{(1,8)} = 4.135$			
	Interaction	No	0.4912	
	$F_{(1,8)} = 0.5203$			

Table S2: List of the statistical results in Figure 2.

Figure	Statistical values	Main effect	*P* value	Post hoc tests
3B1	$Diet_{(SD;HFD)}$	No	0.6892	
	$F_{(1,16)} = 0.166$			
	$Stress_{(Ctrl;CUMS)}$	No	0.2178	
	$F_{(1,16)} = 1.646$			
	Interaction	No	0.5905	
	$F_{(1,16)} = 0.302$			
3B2	$Diet_{(SD;HFD)}$	No	0.3389	
	$F_{(1,16)} = 0.9717$			
	$Stress_{(Ctrl;CUMS)}$	Yes	0.0132	
	$F_{(1,16)} = 7.755$			
	Interaction	No	0.7778	
	$F_{(1,16)} = 0.0824$			
3B3	$Diet_{(SD;HFD)}$	No	0.0634	
	$F_{(1,16)} = 3.979$			
	$Stress_{(Ctrl;CUMS)}$	No	0.1086	
	$F_{(1,16)} = 2.887$			
	Interaction	No	0.7785	
	$F_{(1,16)} = 0.082$			
3B4	$Diet_{(SD;HFD)}$	No	0.2641	
	$F_{(1,16)} = 1.340$			
	$Stress_{(Ctrl;CUMS)}$	Yes	0.0261	
	$F_{(1,16)} = 6.007$			
	Interaction	No	0.4259	
	$F_{(1,16)} = 0.6674$			

(Table continues.)

Figure	Statistical values	Main effect	*P* value	Post hoc tests
3B5-	$Diet_{(SD;HFD)}$	No	0.1930	
a score of 0	$F_{(1,16)} = 1.847$			
	$Stress_{(Ctrl;CUMS)}$	No	0.0653	
	$F_{(1,16)} = 3.916$			
	Interaction	No	0.6034	
	$F_{(1,16)} = 0.2809$			
3B5-	$Diet_{(SD;HFD)}$	No	0.9647	
a score of 1	$F_{(1,16)} = 0.002$			
	$Stress_{(Ctrl;CUMS)}$	No	0.4945	
	$F_{(1,16)} = 0.489$			
	Interaction	No	0.7118	
	$F_{(1,16)} = 0.142$			
3B5-	$Diet_{(SD;HFD)}$	No	0.2494	
a score of 2	$F_{(1,16)} = 1.429$			
	$Stress_{(Ctrl;CUMS)}$	No	0.3912	
	$F_{(1,16)} = 0.777$			
	Interaction	No	0.3086	
	$F_{(1,16)} = 1.106$			
3B5-	$Diet_{(SD;HFD)}$	No	0.8445	
a score of 3	$F_{(1,16)} = 0.0397$			
	$Stress_{(Ctrl;CUMS)}$	No	0.5128	
	$F_{(1,16)} = 0.448$			
	Interaction	No	0.8025	
	$F_{(1,16)} = 0.065$			

(Table continues.)

Figure	Statistical values	Main effect	*P* value	Post hoc tests
3C1	$\text{Diet}_{(SD;HFD)}$	No	0.2837	
	$F_{(1,16)} = 1.231$			
	$\text{Stress}_{(Ctrl;CUMS)}$	No	0.1758	
	$F_{(1,16)} = 2.006$			
	Interaction	No	0.3910	
	$F_{(1,16)} = 0.7775$			
3C2	$\text{Diet}_{(SD;HFD)}$	No	0.6681	
	$F_{(1,16)} = 0.1908$			
	$\text{Stress}_{(Ctrl;CUMS)}$	No	0.9642	
	$F_{(1,16)} = 0.0021$			
	Interaction	No	0.8330	
	$F_{(1,16)} = 0.0459$			
3C3	$\text{Diet}_{(SD;HFD)}$	No	0.0782	
	$F_{(1,16)} = 3.540$			
	$\text{Stress}_{(Ctrl;CUMS)}$	No	0.4768	
	$F_{(1,16)} = 0.5309$			
	Interaction	No	0.8060	
	$F_{(1,16)} = 0.0624$			
3C4	$\text{Diet}_{(SD;HFD)}$	No	0.6224	
	$F_{(1,16)} = 0.2521$			
	$\text{Stress}_{(Ctrl;CUMS)}$	No	0.9157	
	$F_{(1,16)} = 0.0116$			
	Interaction	No	0.8320	
	$F_{(1,16)} = 0.0465$			

(Table continues.)

Figure	Statistical values	Main effect	*P* value	Post hoc tests
3C5-	$Diet_{(SD;HFD)}$	No	0.2100	
a score of 0	$F_{(1,16)} = 1.706$			
	$Stress_{(Ctrl;CUMS)}$	No	0.1620	
	$F_{(1,16)} = 2.150$			
	Interaction	No	0.2487	
	$F_{(1,16)} = 1.433$			
3C5-	$Diet_{(SD;HFD)}$	No	0.8286	
a score of 1	$F_{(1,16)} = 0.048$			
	$Stress_{(Ctrl;CUMS)}$	No	0.6781	
	$F_{(1,16)} = 0.179$			
	Interaction	No	0.9944	
	$F_{(1,16)} < 0.0001$			
3C5-	$Diet_{(SD;HFD)}$	No	0.8282	
a score of 2	$F_{(1,16)} = 0.0487$			
	$Stress_{(Ctrl;CUMS)}$	No	0.2251	
	$F_{(1,16)} = 1.592$			
	Interaction	No	0.3324	
	$F_{(1,16)} = 0.9991$			
3C5-	$Diet_{(SD;HFD)}$	No	0.2022	
a score of 3	$F_{(1,16)} = 1.769$			
	$Stress_{(Ctrl;CUMS)}$	No	0.6041	
	$F_{(1,16)} = 0.2798$			
	Interaction	No	0.9077	
	$F_{(1,16)} = 0.0139$			

(Table continues.)

Figure	Statistical values	Main effect	*P* value	Post hoc tests
3D1	Diet$_{(SD;HFD)}$ $F_{(1,16)} = 2.577$	No	0.1280	
	Stress$_{(Ctrl;CUMS)}$ $F_{(1,16)} = 0.2019$	No	0.6592	
	Interaction $F_{(1,16)} = 0.1112$	No	0.7432	
3D2	Diet$_{(SD;HFD)}$ $F_{(1,16)} = 0.2388$	No	0.6317	
	Stress$_{(Ctrl;CUMS)}$ $F_{(1,16)} = 4.359$	No	0.0532	
	Interaction $F_{(1,16)} = 0.5365$	No	0.4745	
3D3	Diet$_{(SD;HFD)}$ $F_{(1,16)} = 1.889$	No	0.1883	
	Stress$_{(Ctrl;CUMS)}$ $F_{(1,16)} = 6.860$	Yes	0.0186	
	Interaction $F_{(1,16)} = 0.024$	No	0.8780	
3D4	Diet$_{(SD;HFD)}$ $F_{(1,16)} = 1.464$	No	0.2439	
	Stress$_{(Ctrl;CUMS)}$ $F_{(1,16)} = 5.251$	Yes	0.0358	
	Interaction $F_{(1,16)} = 1.296$	No	0.2718	

(Table continues.)

Figure	Statistical values	Main effect	*P* value	Post hoc tests
3D5-	$Diet_{(SD;HFD)}$	No	0.1221	
a score of 0	$F_{(1,16)} = 2.665$			
	$Stress_{(Ctrl;CUMS)}$	No	0.2096	
	$F_{(1,16)} = 1.709$			
	Interaction	No	0.2778	
	$F_{(1,16)} = 1.262$			
3D5-	$Diet_{(SD;HFD)}$	No	0.4671	
a score of 1	$F_{(1,16)} = 0.5551$			
	$Stress_{(Ctrl;CUMS)}$	No	0.6591	
	$F_{(1,16)} = 0.2020$			
	Interaction	No	0.9548	
	$F_{(1,16)} = 0.0033$			
3D5-	$Diet_{(SD;HFD)}$	Yes	0.0370	
a score of 2	$F_{(1,16)} = 5.180$			
	$Stress_{(Ctrl;CUMS)}$	No	0.0728	
	$F_{(1,16)} = 3.689$			
	Interaction	No	0.2331	
	$F_{(1,16)} = 1.536$			
3D5-	$Diet_{(SD;HFD)}$	No	0.1413	
a score of 3	$F_{(1,16)} = 2.395$			
	$Stress_{(Ctrl;CUMS)}$	No	0.4629	
	$F_{(1,16)} = 0.566$			
	Interaction	No	0.6438	
	$F_{(1,16)} = 0.2221$			

(Table continues.)

Figure	Statistical values	Main effect	*P* value	Post hoc tests
3E1	$Diet_{(SD;HFD)}$	No	0.0656	
	$F_{(1,16)} = 3.908$			
	$Stress_{(Ctrl;CUMS)}$	No	0.3151	
	$F_{(1,16)} = 1.075$			
	Interaction	No	0.2559	
	$F_{(1,16)} = 1.388$			
3E2	$Diet_{(SD;HFD)}$	No	0.1062	
	$F_{(1,16)} = 2.930$			
	$Stress_{(Ctrl;CUMS)}$	Yes	0.0054	
	$F_{(1,16)} = 10.35$			
	Interaction	No	0.6614	
	$F_{(1,16)} = 0.1992$			
3E3	$Diet_{(SD;HFD)}$	Yes	0.0155	
	$F_{(1,16)} = 7.329$			
	$Stress_{(Ctrl;CUMS)}$	No	0.0786	
	$F_{(1,16)} = 3.529$			
	Interaction	No	0.0731	
	$F_{(1,16)} = 3.679$			
3E4	$Diet_{(SD;HFD)}$	Yes	0.0141	
	$F_{(1,16)} = 7.583$			
	$Stress_{(Ctrl;CUMS)}$	Yes	0.0043	
	$F_{(1,16)} = 11.08$			
	Interaction	No	0.3586	
	$F_{(1,16)} = 0.8936$			

(Table continues.)

Figure	Statistical values	Main effect	*P* value	Post hoc tests
3E5-<br>a score of 0	Diet$_{(SD;HFD)}$<br>$F_{(1,16)} = 61.54$	Yes	<0.0001	SD-CUMS vs. HFD-CUMS: *P* = 0.0122
	Stress$_{(Ctrl;CUMS)}$<br>$F_{(1,16)} = 12.35$	Yes	0.0029	
	Interaction<br>$F_{(1,16)} = 7.798$	Yes	0.0130	
3E5-<br>a score of 1	Diet$_{(SD;HFD)}$<br>$F_{(1,16)} = 0.8241$	No	0.3774	
	Stress$_{(Ctrl;CUMS)}$<br>$F_{(1,16)} = 0.0040$	No	0.9502	
	Interaction<br>$F_{(1,16)} = 0.1429$	No	0.7104	
3E5-<br>a score of 2	Diet$_{(SD;HFD)}$<br>$F_{(1,16)} = 2.722$	No	0.1185	
	Stress$_{(Ctrl;CUMS)}$<br>$F_{(1,16)} = 0.6169$	No	0.4437	
	Interaction<br>$F_{(1,16)} = 0.1552$	No	0.6988	
3E5-<br>a score of 3	Diet$_{(SD;HFD)}$<br>$F_{(1,16)} = 0.5355$	No	0.4749	
	Stress$_{(Ctrl;CUMS)}$<br>$F_{(1,16)} = 0.7548$	No	0.3978	
	Interaction<br>$F_{(1,16)} = 0.1595$	No	0.6949	

(Table continues.)

Figure	Statistical values	Main effect	*P* value	Post hoc tests
3F1	$Diet_{(SD;HFD)}$	No	0.9942	
	$F_{(1,16)} < 0.0001$			
	$Stress_{(Ctrl;CUMS)}$	Yes	0.0203	
	$F_{(1,16)} = 6.634$			
	Interaction	No	0.7514	
	$F_{(1,16)} = 0.1039$			
3F2	$Diet_{(SD;HFD)}$	No	0.0912	
	$F_{(1,16)} = 3.230$			
	$Stress_{(Ctrl;CUMS)}$	No	0.9905	
	$F_{(1,16)} = 0.0001$			
	Interaction	No	0.9935	
	$F_{(1,16)} < 0.0001$			
3F3	$Diet_{(SD;HFD)}$	Yes	0.0438	
	$F_{(1,16)} = 4.790$			
	$Stress_{(Ctrl;CUMS)}$	No	0.5086	
	$F_{(1,16)} = 0.4572$			
	Interaction	No	0.9467	
	$F_{(1,16)} = 0.0046$			
3F4	$Diet_{(SD;HFD)}$	Yes	0.0008	
	$F_{(1,16)} = 16.79$			
	$Stress_{(Ctrl;CUMS)}$	No	0.7558	
	$F_{(1,16)} = 0.1001$			
	Interaction	No	0.7461	
	$F_{(1,16)} = 0.1085$			

(Table continues.)

Figure	Statistical values	Main effect	*P* value	Post hoc tests
3F5-	$Diet_{(SD;HFD)}$	No	0.0753	
a score of 0	$F_{(1,16)} = 3.617$			
	$Stress_{(Ctrl;CUMS)}$	No	0.3861	
	$F_{(1,16)} = 0.794$			
	Interaction	No	0.8065	
	$F_{(1,16)} = 0.062$			
3F5-	$Diet_{(SD;HFD)}$	No	0.4109	
a score of 1	$F_{(1,16)} = 0.7130$			
	$Stress_{(Ctrl;CUMS)}$	No	0.5044	
	$F_{(1,16)} = 0.4663$			
	Interaction	No	0.6796	
	$F_{(1,16)} = 0.1770$			
3F5-	$Diet_{(SD;HFD)}$	No	0.4172	
a score of 2	$F_{(1,16)} = 0.6935$			
	$Stress_{(Ctrl;CUMS)}$	No	0.7303	
	$F_{(1,16)} = 0.1230$			
	Interaction	No	0.9857	
	$F_{(1,16)} = 0.0003$			
3F5-	$Diet_{(SD;HFD)}$	Yes	0.0323	
a score of 3	$F_{(1,16)} = 5.495$			
	$Stress_{(Ctrl;CUMS)}$	No	0.1051	
	$F_{(1,16)} = 2.951$			
	Interaction	No	0.5763	
	$F_{(1,16)} = 0.3254$			

(Table continues.)

Figure	Statistical values	Main effect	*P* value	Post hoc tests
3G1	$Diet_{(SD;HFD)}$	No	0.2630	
	$F_{(1,16)} = 1.346$			
	$Stress_{(Ctrl;CUMS)}$	No	0.7763	
	$F_{(1,16)} = 0.0835$			
	Interaction	No	0.6392	
	$F_{(1,16)} = 0.2284$			
3G2	$Diet_{(SD;HFD)}$	Yes	0.0042	
	$F_{(1,16)} = 11.13$			
	$Stress_{(Ctrl;CUMS)}$	No	0.1588	
	$F_{(1,16)} = 2.185$			
	Interaction	No	0.5798	
	$F_{(1,16)} = 0.3195$			
3G3	$Diet_{(SD;HFD)}$	No	0.0615	
	$F_{(1,16)} = 4.042$			
	$Stress_{(Ctrl;CUMS)}$	Yes	0.0077	
	$F_{(1,16)} = 9.294$			
	Interaction	No	0.7357	
	$F_{(1,16)} = 0.1180$			
3G4	$Diet_{(SD;HFD)}$	Yes	0.0015	
	$F_{(1,16)} = 14.50$			
	$Stress_{(Ctrl;CUMS)}$	Yes	0.0412	
	$F_{(1,16)} = 4.927$			
	Interaction	No	0.9449	
	$F_{(1,16)} = 0.005$			

(Table continues.)

Figure	Statistical values	Main effect	*P* value	Post hoc tests
3G5-	Diet$_{(SD;HFD)}$	No	0.1676	
a score of 0	$F_{(1,16)} = 2.090$			
	Stress$_{(Ctrl;CUMS)}$	No	0.5088	
	$F_{(1,16)} = 0.4567$			
	Interaction	No	0.8369	
	$F_{(1,16)} = 0.0438$			
3G5-	Diet$_{(SD;HFD)}$	No	0.0911	
a score of 1	$F_{(1,16)} = 3.233$			
	Stress$_{(Ctrl;CUMS)}$	No	0.8572	
	$F_{(1,16)} = 0.033$			
	Interaction	No	0.4596	
	$F_{(1,16)} = 0.5742$			
3G5-	Diet$_{(SD;HFD)}$	No	0.0927	
a score of 2	$F_{(1,16)} = 3.197$			
	Stress$_{(Ctrl;CUMS)}$	No	0.9774	
	$F_{(1,16)} = 0.0008$			
	Interaction	No	0.5930	
	$F_{(1,16)} = 0.2974$			
3G5-	Diet$_{(SD;HFD)}$	Yes	0.0137	
a score of 3	$F_{(1,16)} = 7.673$			
	Stress$_{(Ctrl;CUMS)}$	No	0.2970	
	$F_{(1,16)} = 1.162$			
	Interaction	No	0.3090	
	$F_{(1,16)} = 1.104$			

(Table continues.)

Figure	Statistical values	Main effect	*P* value	Post hoc tests
3I	Diet$_{(SD;HFD)}$ $F_{(1,16)} = 3.240$	No	0.0908	
	Stress$_{(Ctrl;CUMS)}$ $F_{(1,16)} = 9.083$	Yes	0.0082	
	Interaction $F_{(1,16)} = 0.1103$	No	0.7441	
3K	Diet$_{(SD;HFD)}$ $F_{(1,16)} = 72.15$	Yes	<0.0001	SD-CUMS vs. HFD-CUMS: $P < 0.0001$
	Stress$_{(Ctrl;CUMS)}$ $F_{(1,16)} = 17.05$	Yes	0.0008	
	Interaction $F_{(1,16)} = 6.439$	Yes	0.0220	

Table S3: List of the statistical results in Figure 3.

Figure	Statistical values	Main effect	*P* value	Post hoc tests
6D	$\text{Serum}_{(SD;HFD)}$ $F_{(1,8)} = 12.74$	Yes	0.0073	Veh-SD vs. Veh-HFD: *P* = 0.0008
	$\text{Drug}_{(Veh;Enz)}$ $F_{(1,8)} = 115.7$	Yes	<0.0001	Veh-HFD vs. Enz-HFD: *P* < 0.0001
	Interaction $F_{(1,8)} = 33.48$	Yes	0.0004	
6E	$\text{Serum}_{(SD;HFD)}$ $F_{(1,8)} = 11.37$	Yes	0.0098	Veh-SD vs. Veh-HFD: *P* = 0.0038
	$\text{Drug}_{(Veh;Enz)}$ $F_{(1,8)} = 1.03$	No	0.3393	Veh-HFD vs. Enz-HFD: *P* = 0.0335
	Interaction $F_{(1,8)} = 15.36$	Yes	0.0044	
6F	$\text{Serum}_{(SD;HFD)}$ $F_{(1,16)} = 19.92$	Yes	0.0004	Veh-SD vs. Veh-HFD: *P* = 0.0003
	$\text{Drug}_{(Veh;Enz)}$ $F_{(1,16)} = 5.80$	Yes	0.0285	Veh-HFD vs. Enz-HFD: *P* = 0.0057
	Interaction $F_{(1,16)} = 10.07$	Yes	0.0059	
6G	$\text{Serum}_{(SD;HFD)}$ $F_{(1,8)} = 356.1$	Yes	<0.0001	Veh-SD vs. Veh-HFD: *P* < 0.0001
	$\text{Drug}_{(Veh;Enz)}$ $F_{(1,8)} = 42.02$	Yes	0.0002	Veh-HFD vs. Enz-HFD: *P* = 0.0003
	Interaction $F_{(1,8)} = 18.97$	Yes	0.0024	

Table S4: List of the statistical results in Figure 6.

Figure	Statistical values	Main effect	*P* value	Post hoc tests
S5A	Diet$_{(SD;HFD)}$	No	0.6638	
	$F_{(1,32)} = 0.1925$			
	Stress$_{(Ctrl;CUMS)}$	No	0.1926	
	$F_{(1,32)} = 1.771$			
	Interaction	No	0.1241	
	$F_{(1,32)} = 2.494$			
S5B	Diet$_{(SD;HFD)}$	No	0.9404	
	$F_{(1,32)} = 0.0057$			
	Stress$_{(Ctrl;CUMS)}$	No	0.5261	
	$F_{(1,32)} = 0.4108$			
	Interaction	No	0.6406	
	$F_{(1,32)} = 0.2221$			
S5C	Diet$_{(SD;HFD)}$	No	0.5178	
	$F_{(1,32)} = 0.4276$			
	Stress$_{(Ctrl;CUMS)}$	No	0.2440	
	$F_{(1,32)} = 1.409$			
	Interaction	No	0.3826	
	$F_{(1,32)} = 0.7839$			
S5D	Diet$_{(SD;HFD)}$	No	0.3167	
	$F_{(1,32)} = 1.035$			
	Stress$_{(Ctrl;CUMS)}$	No	0.2374	
	$F_{(1,32)} = 1.450$			
	Interaction	No	0.3787	
	$F_{(1,32)} = 0.7969$			

Table S5: List of the statistical results in Figure S5.

Figure	Statistical values	Main effect	*P* value	Post hoc tests
S6D1	$Diet_{(SD;HFD)}$	No	0.1034	
	$F_{(1,8)} = 3.378$			
	$Stress_{(Ctrl;CUMS)}$	No	0.2443	
	$F_{(1,8)} = 1.580$			
	Interaction	No	0.6123	
	$F_{(1,8)} = 0.278$			
S6D2	$Diet_{(SD;HFD)}$	No	0.0534	
	$F_{(1,8)} = 5.123$			
	$Stress_{(Ctrl;CUMS)}$	No	0.0560	
	$F_{(1,8)} = 4.989$			
	Interaction	No	0.7611	
	$F_{(1,8)} = 0.099$			
S6D4	$Diet_{(SD;HFD)}$	No	0.0797	
	$F_{(1,8)} = 4.027$			
	$Stress_{(Ctrl;CUMS)}$	No	0.3694	
	$F_{(1,8)} = 0.904$			
	Interaction	No	0.6410	
	$F_{(1,8)} = 0.235$			
S6D5	$Diet_{(SD;HFD)}$	No	0.5600	
	$F_{(1,8)} = 0.3697$			
	$Stress_{(Ctrl;CUMS)}$	No	0.8927	
	$F_{(1,8)} = 0.0194$			
	Interaction	No	0.9879	
	$F_{(1,8)} = 0.0002$			

(Table continues.)

Figure	Statistical values	Main effect	*P* value	Post hoc tests
S6E1	Diet$_{(SD;HFD)}$	No	0.4697	
	$F_{(1,8)} = 0.5759$			
	Stress$_{(Ctrl;CUMS)}$	No	0.7376	
	$F_{(1,8)} = 0.1203$			
	Interaction	No	0.5515	
	$F_{(1,8)} = 0.3864$			
S6E2	Diet$_{(SD;HFD)}$	No	0.2032	
	$F_{(1,8)} = 1.921$			
	Stress$_{(Ctrl;CUMS)}$	No	0.8573	
	$F_{(1,8)} = 0.0345$			
	Interaction	No	0.9696	
	$F_{(1,8)} = 0.0015$			
S6E4	Diet$_{(SD;HFD)}$	No	0.5042	
	$F_{(1,8)} = 0.4890$			
	Stress$_{(Ctrl;CUMS)}$	No	0.3705	
	$F_{(1,8)} = 0.9004$			
	Interaction	No	0.7072	
	$F_{(1,8)} = 0.1516$			
S6E5	Diet$_{(SD;HFD)}$	No	0.4206	
	$F_{(1,8)} = 0.7207$			
	Stress$_{(Ctrl;CUMS)}$	No	0.2821	
	$F_{(1,8)} = 1.330$			
	Interaction	No	0.9301	
	$F_{(1,8)} = 0.0082$			

(Table continues.)

Figure	Statistical values	Main effect	*P* value	Post hoc tests
S6F1	$Diet_{(SD;HFD)}$	No	0.1265	
	$F_{(1,8)} = 2.909$			
	$Stress_{(Ctrl;CUMS)}$	No	0.6458	
	$F_{(1,8)} = 0.2279$			
	Interaction	No	0.5883	
	$F_{(1,8)} = 0.3179$			
S6F2	$Diet_{(SD;HFD)}$	No	0.2230	
	$F_{(1,8)} = 1.745$			
	$Stress_{(Ctrl;CUMS)}$	No	0.5443	
	$F_{(1,8)} = 0.4009$			
	Interaction	No	0.4472	
	$F_{(1,8)} = 0.6388$			
S6F4	$Diet_{(SD;HFD)}$	No	0.0770	
	$F_{(1,8)} = 4.117$			
	$Stress_{(Ctrl;CUMS)}$	No	0.3694	
	$F_{(1,8)} = 0.9047$			
	Interaction	No	0.7148	
	$F_{(1,8)} = 0.1433$			
S6F5	$Diet_{(SD;HFD)}$	No	0.8135	
	$F_{(1,8)} = 0.0595$			
	$Stress_{(Ctrl;CUMS)}$	No	0.0634	
	$F_{(1,8)} = 4.639$			
	Interaction	No	0.7589	
	$F_{(1,8)} = 0.1009$			

(Table continues.)

Figure	Statistical values	Main effect	*P* value	Post hoc tests
S6G1	$Diet_{(SD;HFD)}$	No	0.0775	
	$F_{(1,8)} = 4.098$			
	$Stress_{(Ctrl;CUMS)}$	No	0.0859	
	$F_{(1,8)} = 3.833$			
	Interaction	No	0.0830	
	$F_{(1,8)} = 3.921$			
S6G2	$Diet_{(SD;HFD)}$	No	0.2387	
	$F_{(1,8)} = 1.621$			
	$Stress_{(Ctrl;CUMS)}$	No	0.4109	
	$F_{(1,8)} = 0.7525$			
	Interaction	No	0.1506	
	$F_{(1,8)} = 2.526$			
S6G4	$Diet_{(SD;HFD)}$	No	0.1559	
	$F_{(1,8)} = 2.454$			
	$Stress_{(Ctrl;CUMS)}$	No	0.1690	
	$F_{(1,8)} = 2.285$			
	Interaction	No	0.0555	
	$F_{(1,8)} = 5.012$			
S6G5	$Diet_{(SD;HFD)}$	No	0.7818	
	$F_{(1,8)} = 0.0821$			
	$Stress_{(Ctrl;CUMS)}$	No	0.8843	
	$F_{(1,8)} = 0.0226$			
	Interaction	No	0.9985	
	$F_{(1,8)} < 0.0001$			

(Table continues.)

Figure	Statistical values	Main effect	*P* value	Post hoc tests
S6H1	$Diet_{(SD;HFD)}$	No	0.1019	
	$F_{(1,8)} = 3.412$			
	$Stress_{(Ctrl;CUMS)}$	No	0.0786	
	$F_{(1,8)} = 4.063$			
	Interaction	No	0.0886	
	$F_{(1,8)} = 3.757$			
S6H2	$Diet_{(SD;HFD)}$	No	0.0886	
	$F_{(1,8)} = 3.756$			
	$Stress_{(Ctrl;CUMS)}$	No	0.0783	
	$F_{(1,8)} = 4.074$			
	Interaction	No	0.2578	
	$F_{(1,8)} = 1.484$			
S6H4	$Diet_{(SD;HFD)}$	No	0.1494	
	$F_{(1,8)} = 2.543$			
	$Stress_{(Ctrl;CUMS)}$	No	0.1618	
	$F_{(1,8)} = 2.376$			
	Interaction	No	0.3759	
	$F_{(1,8)} = 0.879$			
S6H5	$Diet_{(SD;HFD)}$	No	0.9039	
	$F_{(1,8)} = 0.0155$			
	$Stress_{(Ctrl;CUMS)}$	No	0.7321	
	$F_{(1,8)} = 0.1257$			
	Interaction	No	0.9289	
	$F_{(1,8)} = 0.0085$			

(Table continues.)

Figure	Statistical values	Main effect	*P* value	Post hoc tests
S6I1	$\text{Diet}_{(SD;HFD)}$	Yes	0.0457	
	$F_{(1,8)} = 5.586$			
	$\text{Stress}_{(Ctrl;CUMS)}$	No	0.5271	
	$F_{(1,8)} = 0.437$			
	Interaction	No	0.8476	
	$F_{(1,8)} = 0.039$			
S6I2	$\text{Diet}_{(SD;HFD)}$	Yes	0.0224	
	$F_{(1,8)} = 7.973$			
	$\text{Stress}_{(Ctrl;CUMS)}$	No	0.0696	
	$F_{(1,8)} = 4.383$			
	Interaction	No	0.2079	
	$F_{(1,8)} = 1.877$			
S6I4	$\text{Diet}_{(SD;HFD)}$	Yes	0.0436	
	$F_{(1,8)} = 5.732$			
	$\text{Stress}_{(Ctrl;CUMS)}$	No	0.7176	
	$F_{(1,8)} = 0.140$			
	Interaction	No	0.9610	
	$F_{(1,8)} = 0.003$			
S6I5	$\text{Diet}_{(SD;HFD)}$	Yes	0.0006	
	$F_{(1,8)} = 30.27$			
	$\text{Stress}_{(Ctrl;CUMS)}$	No	0.5267	
	$F_{(1,8)} = 0.438$			
	Interaction	No	0.9364	
	$F_{(1,8)} = 0.0068$			

(Table continues.)

Figure	Statistical values	Main effect	*P* value	Post hoc tests
S6J1	$Diet_{(SD;HFD)}$	No	0.8274	
	$F_{(1,8)} = 0.0507$			
	$Stress_{(Ctrl;CUMS)}$	No	0.4170	
	$F_{(1,8)} = 0.7323$			
	Interaction	No	0.8464	
	$F_{(1,8)} = 0.0401$			
S6J2	$Diet_{(SD;HFD)}$	No	0.2568	
	$F_{(1,8)} = 1.492$			
	$Stress_{(Ctrl;CUMS)}$	No	0.7510	
	$F_{(1,8)} = 0.1079$			
	Interaction	No	0.8389	
	$F_{(1,8)} = 0.0441$			
S6J4	$Diet_{(SD;HFD)}$	No	0.8233	
	$F_{(1,8)} = 0.0532$			
	$Stress_{(Ctrl;CUMS)}$	No	0.8831	
	$F_{(1,8)} = 0.0230$			
	Interaction	No	0.7655	
	$F_{(1,8)} = 0.0953$			
S6J5	$Diet_{(SD;HFD)}$	No	0.2322	
	$F_{(1,8)} = 1.671$			
	$Stress_{(Ctrl;CUMS)}$	No	0.6801	
	$F_{(1,8)} = 0.183$			
	Interaction	No	0.7109	
	$F_{(1,8)} = 0.148$			

(Table continues.)

Figure	Statistical values	Main effect	*P* value	Post hoc tests
S6K1	$Diet_{(SD;HFD)}$	No	0.2061	
	$F_{(1,8)} = 1.894$			
	$Stress_{(Ctrl;CUMS)}$	No	0.4321	
	$F_{(1,8)} = 0.6843$			
	Interaction	No	0.5408	
	$F_{(1,8)} = 0.4080$			
S6K2	$Diet_{(SD;HFD)}$	No	0.2030	
	$F_{(1,8)} = 1.922$			
	$Stress_{(Ctrl;CUMS)}$	No	0.6571	
	$F_{(1,8)} = 0.2124$			
	Interaction	No	0.9099	
	$F_{(1,8)} = 0.0136$			
S6K4	$Diet_{(SD;HFD)}$	No	0.1725	
	$F_{(1,8)} = 2.244$			
	$Stress_{(Ctrl;CUMS)}$	No	0.8303	
	$F_{(1,8)} = 0.0491$			
	Interaction	No	0.2496	
	$F_{(1,8)} = 1.541$			
S6K5	$Diet_{(SD;HFD)}$	No	0.6298	
	$F_{(1,8)} = 0.2510$			
	$Stress_{(Ctrl;CUMS)}$	No	0.7843	
	$F_{(1,8)} = 0.0801$			
	Interaction	No	0.8482	
	$F_{(1,8)} = 0.0391$			

(Table continues.)

Figure	Statistical values	Main effect	*P* value	Post hoc tests
S6L1	$Diet_{(SD;HFD)}$	No	0.2186	
	$F_{(1,8)} = 1.782$			
	$Stress_{(Ctrl;CUMS)}$	No	0.1902	
	$F_{(1,8)} = 2.049$			
	Interaction	No	0.2255	
	$F_{(1,8)} = 1.725$			
S6L2	$Diet_{(SD;HFD)}$	No	0.0779	
	$F_{(1,8)} = 4.085$			
	$Stress_{(Ctrl;CUMS)}$	No	0.1081	
	$F_{(1,8)} = 3.272$			
	Interaction	No	0.0535	
	$F_{(1,8)} = 5.120$			
S6L4	$Diet_{(SD;HFD)}$	No	0.0706	
	$F_{(1,8)} = 4.347$			
	$Stress_{(Ctrl;CUMS)}$	No	0.0914	
	$F_{(1,8)} = 3.679$			
	Interaction	No	0.1741	
	$F_{(1,8)} = 2.226$			
S6L5	$Diet_{(SD;HFD)}$	No	0.5817	
	$F_{(1,8)} = 0.3296$			
	$Stress_{(Ctrl;CUMS)}$	No	0.5101	
	$F_{(1,8)} = 0.4752$			
	Interaction	No	0.6461	
	$F_{(1,8)} = 0.2276$			

(Table continues.)

Figure	Statistical values	Main effect	*P* value	Post hoc tests
S6M1	Diet$_{(SD;HFD)}$	No	0.1007	
	$F_{(1,8)} = 3.442$			
	Stress$_{(Ctrl;CUMS)}$	No	0.4947	
	$F_{(1,8)} = 0.512$			
	Interaction	No	0.1427	
	$F_{(1,8)} = 2.642$			
S6M2	Diet$_{(SD;HFD)}$	No	0.1121	
	$F_{(1,8)} = 3.185$			
	Stress$_{(Ctrl;CUMS)}$	No	0.2659	
	$F_{(1,8)} = 1.431$			
	Interaction	No	0.2351	
	$F_{(1,8)} = 1.648$			
S6M4	Diet$_{(SD;HFD)}$	No	0.0967	
	$F_{(1,8)} = 3.540$			
	Stress$_{(Ctrl;CUMS)}$	No	0.8499	
	$F_{(1,8)} = 0.038$			
	Interaction	No	0.0574	
	$F_{(1,8)} = 4.916$			
S6M5	Diet$_{(SD;HFD)}$	No	0.0710	
	$F_{(1,8)} = 4.329$			
	Stress$_{(Ctrl;CUMS)}$	No	0.2227	
	$F_{(1,8)} = 1.748$			
	Interaction	No	0.5113	
	$F_{(1,8)} = 0.472$			

Table S6: List of the statistical results in Figure S6.

Figure	Statistical values	Main effect	*P* value	Post hoc tests
S7B1	$Diet_{(SD;HFD)}$	No	0.5622	
	$F_{(1,16)} = 0.3503$			
	$Stress_{(Ctrl;CUMS)}$	No	0.1933	
	$F_{(1,16)} = 1.844$			
	Interaction	No	0.2898	
	$F_{(1,16)} = 1.198$			
S7B2	$Diet_{(SD;HFD)}$	No	0.7430	
	$F_{(1,16)} = 0.1113$			
	$Stress_{(Ctrl;CUMS)}$	No	0.2623	
	$F_{(1,16)} = 1.350$			
	Interaction	No	0.9593	
	$F_{(1,16)} = 0.0027$			
S7B3	$Diet_{(SD;HFD)}$	No	0.2760	
	$F_{(1,16)} = 1.272$			
	$Stress_{(Ctrl;CUMS)}$	No	0.6793	
	$F_{(1,16)} = 0.1773$			
	Interaction	No	0.9671	
	$F_{(1,16)} = 0.0018$			
S7B4	$Diet_{(SD;HFD)}$	No	0.2918	
	$F_{(1,16)} = 1.188$			
	$Stress_{(Ctrl;CUMS)}$	No	0.1991	
	$F_{(1,16)} = 1.794$			
	Interaction	No	0.9099	
	$F_{(1,16)} = 0.0132$			

(Table continues.)

Figure	Statistical values	Main effect	*P* value	Post hoc tests
S7B5-	$Diet_{(SD;HFD)}$	No	0.1308	
a score of 0	$F_{(1,16)} = 2.537$			
	$Stress_{(Ctrl;CUMS)}$	No	0.6969	
	$F_{(1,16)} = 0.1573$			
	Interaction	No	0.4516	
	$F_{(1,16)} = 0.5953$			
S7B5-	$Diet_{(SD;HFD)}$	No	0.9735	
a score of 1	$F_{(1,16)} = 0.0011$			
	$Stress_{(Ctrl;CUMS)}$	No	0.3365	
	$F_{(1,16)} = 0.9819$			
	Interaction	No	0.6077	
	$F_{(1,16)} = 0.2742$			
S7B5-	$Diet_{(SD;HFD)}$	No	0.4981	
a score of 2	$F_{(1,16)} = 0.4805$			
	$Stress_{(Ctrl;CUMS)}$	No	0.2673	
	$F_{(1,16)} = 1.321$			
	Interaction	No	0.3926	
	$F_{(1,16)} = 0.7721$			
S7B5-	$Diet_{(SD;HFD)}$	No	0.1391	
a score of 3	$F_{(1,16)} = 2.423$			
	$Stress_{(Ctrl;CUMS)}$	No	0.5413	
	$F_{(1,16)} = 0.3896$			
	Interaction	No	0.2890	
	$F_{(1,16)} = 1.203$			

(Table continues.)

Figure	Statistical values	Main effect	*P* value	Post hoc tests
S7C1	$Diet_{(SD;HFD)}$	No	0.1452	
	$F_{(1,16)} = 2.346$			
	$Stress_{(Ctrl;CUMS)}$	No	0.3440	
	$F_{(1,16)} = 0.9508$			
	Interaction	No	0.7882	
	$F_{(1,16)} = 0.0746$			
S7C2	$Diet_{(SD;HFD)}$	No	0.3430	
	$F_{(1,16)} = 0.9550$			
	$Stress_{(Ctrl;CUMS)}$	No	0.6424	
	$F_{(1,16)} = 0.2240$			
	Interaction	No	0.2732	
	$F_{(1,16)} = 1.288$			
S7C3	$Diet_{(SD;HFD)}$	No	0.1087	
	$F_{(1,16)} = 2.886$			
	$Stress_{(Ctrl;CUMS)}$	No	0.4164	
	$F_{(1,16)} = 0.696$			
	Interaction	No	0.5487	
	$F_{(1,16)} = 0.3754$			
S7C4	$Diet_{(SD;HFD)}$	No	0.0944	
	$F_{(1,16)} = 3.161$			
	$Stress_{(Ctrl;CUMS)}$	No	0.4758	
	$F_{(1,16)} = 0.533$			
	Interaction	No	0.6598	
	$F_{(1,16)} = 0.2012$			

(Table continues.)

Figure	Statistical values	Main effect	*P* value	Post hoc tests
S7C5-	$Diet_{(SD;HFD)}$	No	0.0779	
a score of 0	$F_{(1,16)} = 3.549$			
	$Stress_{(Ctrl;CUMS)}$	No	0.1361	
	$F_{(1,16)} = 2.463$			
	Interaction	No	0.7561	
	$F_{(1,16)} = 0.100$			
S7C5-	$Diet_{(SD;HFD)}$	No	0.9691	
a score of 1	$F_{(1,16)} = 0.0016$			
	$Stress_{(Ctrl;CUMS)}$	No	0.1708	
	$F_{(1,16)} = 2.057$			
	Interaction	No	0.6932	
	$F_{(1,16)} = 0.1614$			
S7C5-	$Diet_{(SD;HFD)}$	No	0.2535	
a score of 2	$F_{(1,16)} = 1.403$			
	$Stress_{(Ctrl;CUMS)}$	No	0.6058	
	$F_{(1,16)} = 0.2772$			
	Interaction	No	0.7591	
	$F_{(1,16)} = 0.0973$			
S7C5-	$Diet_{(SD;HFD)}$	No	0.7107	
a score of 3	$F_{(1,16)} = 0.1426$			
	$Stress_{(Ctrl;CUMS)}$	No	0.3362	
	$F_{(1,16)} = 0.9831$			
	Interaction	No	0.8859	
	$F_{(1,16)} = 0.0213$			

(Table continues.)

Figure	Statistical values	Main effect	*P* value	Post hoc tests
S7D1	$Diet_{(SD;HFD)}$	No	0.9722	
	$F_{(1,16)} = 0.0013$			
	$Stress_{(Ctrl;CUMS)}$	No	0.3070	
	$F_{(1,16)} = 1.114$			
	Interaction	No	0.5921	
	$F_{(1,16)} = 0.2989$			
S7D2	$Diet_{(SD;HFD)}$	No	0.4052	
	$F_{(1,16)} = 0.7310$			
	$Stress_{(Ctrl;CUMS)}$	No	0.6098	
	$F_{(1,16)} = 0.2709$			
	Interaction	No	0.9909	
	$F_{(1,16)} = 0.0001$			
S7D3	$Diet_{(SD;HFD)}$	No	0.1407	
	$F_{(1,16)} = 2.402$			
	$Stress_{(Ctrl;CUMS)}$	No	0.1117	
	$F_{(1,16)} = 2.834$			
	Interaction	No	0.2041	
	$F_{(1,16)} = 1.753$			
S7D4	$Diet_{(SD;HFD)}$	No	0.0971	
	$F_{(1,16)} = 3.106$			
	$Stress_{(Ctrl;CUMS)}$	No	0.1143	
	$F_{(1,16)} = 2.790$			
	Interaction	No	0.7205	
	$F_{(1,16)} = 0.1326$			

(Table continues.)

Figure	Statistical values	Main effect	*P* value	Post hoc tests
S7D5-	$Diet_{(SD;HFD)}$	No	0.3811	
a score of 0	$F_{(1,16)} = 0.8114$			
	$Stress_{(Ctrl;CUMS)}$	No	0.1750	
	$F_{(1,16)} = 2.014$			
	Interaction	No	0.6229	
	$F_{(1,16)} = 0.2514$			
S7D5-	$Diet_{(SD;HFD)}$	No	0.9746	
a score of 1	$F_{(1,16)} = 0.0010$			
	$Stress_{(Ctrl;CUMS)}$	No	0.3828	
	$F_{(1,16)} = 0.8055$			
	Interaction	No	0.5739	
	$F_{(1,16)} = 0.3296$			
S7D5-	$Diet_{(SD;HFD)}$	No	0.4501	
a score of 2	$F_{(1,16)} = 0.5994$			
	$Stress_{(Ctrl;CUMS)}$	No	0.9225	
	$F_{(1,16)} = 0.0098$			
	Interaction	No	0.8585	
	$F_{(1,16)} = 0.0328$			
S7D5-	$Diet_{(SD;HFD)}$	No	0.1119	
a score of 3	$F_{(1,16)} = 2.831$			
	$Stress_{(Ctrl;CUMS)}$	No	0.1937	
	$F_{(1,16)} = 1.841$			
	Interaction	No	0.5241	
	$F_{(1,16)} = 0.424$			

(Table continues.)

Figure	Statistical values	Main effect	*P* value	Post hoc tests
S7E1	$Diet_{(SD;HFD)}$	No	0.9326	
	$F_{(1,16)} = 0.0074$			
	$Stress_{(Ctrl;CUMS)}$	No	0.9938	
	$F_{(1,16)} < 0.0001$			
	Interaction	No	0.9277	
	$F_{(1,16)} = 0.0085$			
S7E2	$Diet_{(SD;HFD)}$	No	0.8389	
	$F_{(1,16)} = 0.0427$			
	$Stress_{(Ctrl;CUMS)}$	No	0.6292	
	$F_{(1,16)} = 0.2423$			
	Interaction	No	0.9331	
	$F_{(1,16)} = 0.0073$			
S7E3	$Diet_{(SD;HFD)}$	Yes	0.0087	
	$F_{(1,16)} = 8.938$			
	$Stress_{(Ctrl;CUMS)}$	No	0.1401	
	$F_{(1,16)} = 2.411$			
	Interaction	No	0.1570	
	$F_{(1,16)} = 2.205$			
S7E4	$Diet_{(SD;HFD)}$	No	0.2707	
	$F_{(1,16)} = 1.302$			
	$Stress_{(Ctrl;CUMS)}$	No	0.4135	
	$F_{(1,16)} = 0.7050$			
	Interaction	No	0.9466	
	$F_{(1,16)} = 0.0046$			

(Table continues.)

Figure	Statistical values	Main effect	*P* value	Post hoc tests
S7E5-	$Diet_{(SD;HFD)}$	Yes	0.0210	
a score of 0	$F_{(1,16)} = 6.546$			
	$Stress_{(Ctrl;CUMS)}$	No	0.1881	
	$F_{(1,16)} = 1.891$			
	Interaction	No	0.6734	
	$F_{(1,16)} = 0.1843$			
S7E5-	$Diet_{(SD;HFD)}$	No	0.8351	
a score of 1	$F_{(1,16)} = 0.0448$			
	$Stress_{(Ctrl;CUMS)}$	No	0.2278	
	$F_{(1,16)} = 1.573$			
	Interaction	No	0.0840	
	$F_{(1,16)} = 3.396$			
S7E5-	$Diet_{(SD;HFD)}$	No	0.1985	
a score of 2	$F_{(1,16)} = 1.799$			
	$Stress_{(Ctrl;CUMS)}$	No	0.1130	
	$F_{(1,16)} = 2.812$			
	Interaction	No	0.0633	
	$F_{(1,16)} = 3.983$			
S7E5-	$Diet_{(SD;HFD)}$	No	0.2989	
a score of 3	$F_{(1,16)} = 1.153$			
	$Stress_{(Ctrl;CUMS)}$	No	0.1385	
	$F_{(1,16)} = 2.431$			
	Interaction	No	0.9418	
	$F_{(1,16)} = 0.0055$			

(Table continues.)

Figure	Statistical values	Main effect	*P* value	Post hoc tests
S7F1	$Diet_{(SD;HFD)}$	No	0.8945	
	$F_{(1,16)} = 0.0181$			
	$Stress_{(Ctrl;CUMS)}$	No	0.7417	
	$F_{(1,16)} = 0.1125$			
	Interaction	No	0.3852	
	$F_{(1,16)} = 0.7970$			
S7F2	$Diet_{(SD;HFD)}$	No	0.6865	
	$F_{(1,16)} = 0.1690$			
	$Stress_{(Ctrl;CUMS)}$	No	0.4261	
	$F_{(1,16)} = 0.6671$			
	Interaction	No	0.8058	
	$F_{(1,16)} = 0.0625$			
S7F3	$Diet_{(SD;HFD)}$	No	0.1037	
	$F_{(1,16)} = 2.977$			
	$Stress_{(Ctrl;CUMS)}$	No	0.5306	
	$F_{(1,16)} = 0.411$			
	Interaction	No	0.9396	
	$F_{(1,16)} = 0.006$			
S7F4	$Diet_{(SD;HFD)}$	No	0.5472	
	$F_{(1,16)} = 0.3783$			
	$Stress_{(Ctrl;CUMS)}$	No	0.5596	
	$F_{(1,16)} = 0.3550$			
	Interaction	No	0.9774	
	$F_{(1,16)} = 0.0008$			

(Table continues.)

Figure	Statistical values	Main effect	*P* value	Post hoc tests
S7F5-	$Diet_{(SD;HFD)}$	No	0.3689	
a score of 0	$F_{(1,16)} = 0.8549$			
	$Stress_{(Ctrl;CUMS)}$	No	0.7794	
	$F_{(1,16)} = 0.0811$			
	Interaction	No	0.8479	
	$F_{(1,16)} = 0.0380$			
S7F5-	$Diet_{(SD;HFD)}$	No	0.4920	
a score of 1	$F_{(1,16)} = 0.4946$			
	$Stress_{(Ctrl;CUMS)}$	No	0.0563	
	$F_{(1,16)} = 4.236$			
	Interaction	No	0.5023	
	$F_{(1,16)} = 0.4711$			
S7F5-	$Diet_{(SD;HFD)}$	No	0.7004	
a score of 2	$F_{(1,16)} = 0.1535$			
	$Stress_{(Ctrl;CUMS)}$	No	0.0829	
	$F_{(1,16)} = 3.422$			
	Interaction	No	0.2230	
	$F_{(1,16)} = 1.608$			
S7F5-	$Diet_{(SD;HFD)}$	No	0.3921	
a score of 3	$F_{(1,16)} = 0.7735$			
	$Stress_{(Ctrl;CUMS)}$	No	0.1748	
	$F_{(1,16)} = 2.017$			
	Interaction	No	0.7458	
	$F_{(1,16)} = 0.1088$			

(Table continues.)

Figure	Statistical values	Main effect	*P* value	Post hoc tests
S7G1	$Diet_{(SD;HFD)}$	No	0.2306	
	$F_{(1,16)} = 1.553$			
	$Stress_{(Ctrl;CUMS)}$	No	0.0537	
	$F_{(1,16)} = 4.335$			
	Interaction	No	0.6985	
	$F_{(1,16)} = 0.1556$			
S7G2	$Diet_{(SD;HFD)}$	No	0.1865	
	$F_{(1,16)} = 1.905$			
	$Stress_{(Ctrl;CUMS)}$	No	0.0701	
	$F_{(1,16)} = 3.768$			
	Interaction	No	0.8102	
	$F_{(1,16)} = 0.060$			
S7G3	$Diet_{(SD;HFD)}$	No	0.0636	
	$F_{(1,16)} = 3.972$			
	$Stress_{(Ctrl;CUMS)}$	No	0.0710	
	$F_{(1,16)} = 3.740$			
	Interaction	No	0.9226	
	$F_{(1,16)} = 0.010$			
S7G4	$Diet_{(SD;HFD)}$	No	0.0775	
	$F_{(1,16)} = 3.559$			
	$Stress_{(Ctrl;CUMS)}$	No	0.0506	
	$F_{(1,16)} = 4.467$			
	Interaction	No	0.8643	
	$F_{(1,16)} = 0.030$			

(Table continues.)

Figure	Statistical values	Main effect	*P* value	Post hoc tests
S7G5-	$Diet_{(SD;HFD)}$	No	0.5414	
a score of 0	$F_{(1,16)} = 0.3895$			
	$Stress_{(Ctrl;CUMS)}$	No	0.1465	
	$F_{(1,16)} = 2.329$			
	Interaction	No	0.5149	
	$F_{(1,16)} = 0.4436$			
S7G5-	$Diet_{(SD;HFD)}$	No	0.9900	
a score of 1	$F_{(1,16)} = 0.0002$			
	$Stress_{(Ctrl;CUMS)}$	No	0.6688	
	$F_{(1,16)} = 0.1899$			
	Interaction	No	0.3896	
	$F_{(1,16)} = 0.7821$			
S7G5-	$Diet_{(SD;HFD)}$	No	0.3217	
a score of 2	$F_{(1,16)} = 1.046$			
	$Stress_{(Ctrl;CUMS)}$	No	0.2489	
	$F_{(1,16)} = 1.432$			
	Interaction	No	0.3970	
	$F_{(1,16)} = 0.758$			
S7G5-	$Diet_{(SD;HFD)}$	No	0.5523	
a score of 3	$F_{(1,16)} = 0.3686$			
	$Stress_{(Ctrl;CUMS)}$	No	0.1572	
	$F_{(1,16)} = 2.202$			
	Interaction	No	0.5307	
	$F_{(1,16)} = 0.411$			

Table S7: List of the statistical results in Figure S7.

Figure	Statistical values	Main effect	*P* value	Post hoc tests
S10A	$Diet_{(SD;HFD)}$	Yes	<0.0001	
P49	$F_{(1,32)} = 20.70$			
	$Stress_{(Ctrl;CUMS)}$	Yes	0.0225	
	$F_{(1,32)} = 5.75$			
	Interaction	No	0.7367	
	$F_{(1,32)} = 0.12$			
S10A	$Diet_{(SD;HFD)}$	Yes	<0.0001	
P56	$F_{(1,32)} = 24.78$			
	$Stress_{(Ctrl;CUMS)}$	Yes	0.0201	
	$F_{(1,32)} = 5.99$			
	Interaction	No	0.6415	
	$F_{(1,32)} = 0.22$			
S10B	$Diet_{(SD;HFD)}$	Yes	<0.0001	
	$F_{(1,32)} = 54.76$			
	$Stress_{(Ctrl;CUMS)}$	Yes	0.0008	
	$F_{(1,32)} = 13.69$			
	Interaction	No	0.1466	
	$F_{(1,32)} = 2.21$			
S10C	$Diet_{(SD;HFD)}$	No	0.1539	
	$F_{(1,8)} = 2.48$			
	$Stress_{(Ctrl;CUMS)}$	No	0.4468	
	$F_{(1,8)} = 0.64$			
	Interaction	No	0.8500	
	$F_{(1,8)} = 0.04$			

(Table continues.)

Figure	Statistical values	Main effect	*P* value	Post hoc tests
S10D	Diet(SD;HFD)	Yes	0.0028	
	$F_{(1,8)} = 18.00$			
	Stress(Ctrl;CUMS)	No	0.4837	
	$F_{(1,8)} = 0.54$			
	Interaction	No	0.9393	
	$F_{(1,8)} < 0.01$			
S10E	Diet(SD;HFD)	Yes	0.0002	
P49	$F_{(1,32)} = 17.29$			
	Stress(Ctrl;CUMS)	Yes	0.0087	
	$F_{(1,32)} = 7.81$			
	Interaction	No	0.5893	
	$F_{(1,32)} = 0.30$			
S10E	Diet(SD;HFD)	Yes	<0.0001	
P56	$F_{(1,32)} = 30.65$			
	Stress(Ctrl;CUMS)	Yes	0.0017	
	$F_{(1,32)} = 11.74$			
	Interaction	No	0.9583	
	$F_{(1,32)} < 0.01$			
S10F	Diet(SD;HFD)	Yes	<0.0001	
	$F_{(1,32)} = 28.17$			
	Stress(Ctrl;CUMS)	Yes	0.0113	
	$F_{(1,32)} = 7.22$			
	Interaction	No	0.1939	
	$F_{(1,32)} = 1.76$			

(Table continues.)

Figure	Statistical values	Main effect	*P* value	Post hoc tests
S10G	Diet$_{(SD;HFD)}$	No	0.1106	
	$F_{(1,8)} = 3.22$			
	Stress$_{(Ctrl;CUMS)}$	No	0.2201	
	$F_{(1,8)} = 1.77$			
	Interaction	No	0.6404	
	$F_{(1,8)} = 0.24$			
S10H	Diet$_{(SD;HFD)}$	Yes	0.0007	
	$F_{(1,8)} = 28.51$			
	Stress$_{(Ctrl;CUMS)}$	No	0.1827	
	$F_{(1,8)} = 2.13$			
	Interaction	No	0.5027	
	$F_{(1,8)} = 0.49$			

Table S8: List of the statistical results in Figure S10.

References

1. Paxinos G, Franklin KB: **The Mouse Brain in Stereotaxic Coordinates. Third.** Academic Press; 2008.
